# Multiple rare genetic variants co‐segregating with familial IgA nephropathy all act within a single immune‐related network

**DOI:** 10.1111/joim.12565

**Published:** 2016-10-11

**Authors:** S. N. Cox, F. Pesce, J.S. El‐Sayed Moustafa, F. Sallustio, G. Serino, C. Kkoufou, A. Giampetruzzi, N. Ancona, M. Falchi, F. P. Schena, the European IgAN Consortium

**Affiliations:** ^1^ Department of Emergency and Organ TransplantationUniversity of Bari Aldo MoroBariItaly; ^2^ C.A.R.S.O. ConsortiumUniversity of BariBariItaly; ^3^ Department of Genomics of Common DiseaseImperial College LondonLondonUK; ^4^ Department of Twin Research and Genetic EpidemiologyKing's College LondonLondonUK; ^5^ IRCCS ‘de Bellis’Laboratory of Experimental ImmunopathologyBariItaly; ^6^ Department of Soil, Plant and Food SciencesUniversity of Bari Aldo MoroBariItaly; ^7^ ISSIA, CNRBariItaly

**Keywords:** family medicine, gene polymorphism, genetics, glomerulonephritis, kidney disease

## Abstract

**Background:**

IgA nephropathy (IgAN) is a common complex disease with a strong genetic involvement. We aimed to identify novel, rare, highly penetrant risk variants combining family‐based linkage analysis with whole‐exome sequencing (WES).

**Methods:**

Linkage analysis of 16 kindreds of South Italian ancestry was performed using an ‘affected‐only’ strategy. Eight most informative trios composed of two familial cases and an intrafamilial control were selected for WES. High‐priority variants in linked regions were identified and validated using Sanger sequencing. Custom TaqMan assays were designed and carried out in the 16 kindreds and an independent cohort of 240 IgAN patients and 113 control subjects.

**Results:**

We found suggestive linkage signals in 12 loci. After sequential filtering and validation of WES data, we identified 24 private or extremely rare (MAF <0.0003) linked variants segregating with IgAN status. These were present within coding or regulatory regions of 23 genes that merged into a common functional network. The genes were interconnected by *AKT, CTNNB1, NFKB, MYC* and *UBC*, key modulators of WNT/β‐catenin and PI3K/Akt pathways, which are implicated in IgAN pathogenesis. Overlaying publicly available expression data, genes/proteins with expression notably altered in IgAN were included in this immune‐related network. In particular, the network included the glucocorticoid receptor gene, *NR3C1*, which is the target of corticosteroid therapy routinely used in the treatment of IgAN.

**Conclusion:**

Our findings suggest that disease susceptibility could be influenced by multiple rare variants acting in a common network that could provide the starting point for the identification of potential drug targets for personalized therapy.

## Introduction

Immunoglobulin A nephropathy (IgAN) is the most common form of primary glomerulonephritis worldwide amongst patients undergoing renal biopsy 1, 2. The gold standard for diagnosis depends entirely on this invasive procedure. The genetic component of IgAN, supported by familial clustering, may influence the disease on various levels and may also contribute to the variation observed in prevalence 3. The phenotypic variability of IgAN, characterized by recurrent episodes of gross haematuria concomitant with upper respiratory tract infections or persistent microscopic haematuria and/or mild proteinuria, suggests that this glomerulonephritis is a complex disease in which multiple genes may be involved and the kidneys are simple bystanders 2.

Various genomewide genetic approaches have been used to study IgAN with the aim to identify specific markers involved in its susceptibility. Genome‐wide association studies (GWASs) testing common variants with relatively small effects 4 have led to the identification of various susceptibility loci 5, 6, 7, 8, 9. Furthermore, these studies have provided insights into the complex genetic architecture of IgAN and attempted to explain how susceptibility loci correlate with differences in disease prevalence amongst world populations 8, 10. Most of the identified loci have been rigorously replicated suggesting a strong genetic component, but still a large proportion of disease risk remains unexplained and there are likely to be additional loci that have not been discovered 11.

On the other hand, genomewide linkage analyses (GWLAs), evaluating the co‐segregation of genetic markers with the disease in multiplex families, have identified several linked chromosomal regions including 2q36, 4q26–31, 6q22–23 and 17q12–22 12, 13, 14. These loci are distinct from those identified by GWASs and ultimately both approaches have been unable to identify the exact casual genes involved in IgAN susceptibility 15.

Rare variants, which are not interrogated by GWASs, could be responsible for a substantial proportion of complex human diseases 16 and may explain a considerable portion of their missing heritability 4. This concept has been recently demonstrated in an innovatively designed study of age‐related macular degeneration in which rare causative coding variants were pinpointed within known associated genetic loci 17. Because 85% of the disease‐causing mutations are likely to be located in well‐annotated coding regions of the genome 18, the cost‐effective whole‐exome sequencing (WES) strategy is a useful approach in the identification of rare causative variants.

Here, we report the first study of IgAN employing a combined multistep approach for the identification of rare variants associated with this disease. Using the family‐based GWLA approach, we first identified genomic regions containing potential susceptibility loci. Then, WES of selected IgAN familial cases and controls was used to fine map the linked regions and identify rare segregating functional genomic variants potentially involved in the pathogenesis of familial IgAN.

## Materials and methods

### Sample donors

The genomewide linkage analysis involved 34 biopsy‐proven familial IgAN patients and 112 relatives from 16 Italian kindreds of South Italian ancestry (Table 1 and Figure S1). Recruitment strategies are available on the European IgAN Consortium website (www.igan.net) 19. Briefly, familial IgAN was diagnosed when at least two family members had biopsy‐proven IgAN; the remaining family members underwent urinalysis. Unaffected family members had at least three documented normal urinalyses (Figure S1). An independent cohort of 240 biopsy‐proven IgAN patients and 113 healthy blood donors (HBDs) were included in the study for custom TaqMan SNP genotyping assays. Written informed consent was obtained from all study participants. The study was carried out according to the principles of the Declaration of Helsinki and was approved by the Policlinico di Bari Ethics Review Board.

**Table 1:** Clinical and demographic features of IgAN patients, relatives and HBD included in the study^a^

	IgAN^b^	Relatives^b^	IgAN^c^	HBD^c^
Number	34	112	240	113
Male/female	23/11	46/66	164/76	84/29
Age of onset (years)	25 ± 11	n.d	27 ± 11	n.d
sCr (mg/dL)	1.36 ± 0.98	n.d	1.18 ± 0.59	0.87 ± 0.01
eGFR(mL min^−1^ per 1.73 m^2^)	88.3 ± 18	n.d	88 ± 29	n.d
CKD stage 1 (%)	55	n.d	53	n.d
CKD stage 2 (%)	24	n.d	29	n.d
CKD stage 3 (%)	15	n.d	12	n.d
CKD stage 4 (%)	3	n.d	6	n.d
CKD stage 5 (%)	3	n.d	0	n.d
Proteinuria (g/24 h)	1.09 ± 0.75	n.d	1.07 ± 1.28	n.d
Hypertension (%)	37%	n.d	36%	n.d
Histological classification (%)	G1 41% G2 24% G3 35%	n.d	G1 38% G2 41% G3 20%	n.d

a Values are expressed as mean±S.D. Abbreviations: sCr, serum creatinine; eGFR, estimated glomerular filtration rate (eGFR) has been calculated using the CKD‐EPI creatinine formula (mL min^−1^ per 1.73 m^2^); G1, Grade 1 mild; G2, Grade 2 moderate; G3 Grade 3 severe; HBS, Healthy Blood Donors; n.d, not determined. Demographic, clinical and histological data refer to the time of biopsy‐proven diagnosis.

b These patients and relatives have been included in the in the Microarray Genotyping cohort and in the exome‐sequencing study.

c These patients and controls have been included TaqMan genotyping cohort.

### GWLAs of IgAN families

Microarray genotyping is available under the GEO accession number GSE44974 (http://www.ncbi.nlm.nih.gov/geo/) 20, and a detailed description can be found in the Supplementary Methods. Individuals with more than 5% missing genotypes and more than 5% Mendelian errors were excluded. Markers that failed the Hardy–Weinberg equilibrium test (*P* ≤ 1 × 10^−6^) and those with a minor allele frequency (MAF) of ≤0.05 were excluded. Genotyping errors were also detected and removed using Merlin error detection analysis (–error option). Nonparametric linkage (modelling linkage disequilibrium with *r* ^2^ > 0.10) analysis was performed on 146 individuals for a total of 16 families and 227 114 SNPs.

The Whittemore and Halpern NPL all statistics 21 was used to test for allele sharing amongst affected individuals, calculating the LOD score using the Kong and Cox linear model (–npl option) 22. A *P*‐value <5.0 × 10^−3^ (LOD >1.5) 23 was deemed significant. The study was powered to detect a maximum possible LOD score of 5.052 (*P* = 7.05 × 10^−7^) for this data set. The analyses were carried out using Merlin software (version 1.1.2) 24.

### WES of IgAN families

We performed WES on 16 most informative IgAN patients belonging to eight nonconsanguineous families and eight intrafamilial controls. For the selection of the internal (intrafamilial) negative controls, we performed an identical by descent (IBD) analysis on each of these eight families and identified the closest relative (for each affected individual) with the least IBD sharing (genetically discordant) in the region of interest 24, 25. Exome libraries were prepared using 3 μg of genomic DNA. Sequence reads were mapped to the reference human genome (UCSC Genome Browser hg19) using the Burrows–Wheeler aligner (BWA; version 0.5.9‐r16) 26, with default parameters. The Best Practices Workflow of Genome Analysis Toolkit (GATK, http://www.broadinstitute.org) 27 was used for improving the alignments and for genotype calling with recommended parameters. Genotypes were called at first with the GATK Unified Genotyper (UG, version 2.7‐4), and the GATK VariantRecalibrator tool was used to score variant calls by a machine‐learning algorithm and to identify a set of high‐quality variants using the variant quality score recalibration (VQSR) procedure. GATK was used to filter high‐quality variants with hard filtering criteria (variant confidence score ≥30, mapping quality ≥40, read depth ≥5 and strand bias FS filter <60). At a later date, our exome data were also reprocessed using the newer GATK algorithm Haplotype Caller (HC, version v3.3). To be conservative, we decided to retain and evaluate both variant lists generated by HC and UG. These two tools are based on different algorithms 28, but both evaluate haplotypes using an affine gap penalty pair hidden Markov model 29. Variants were then annotated with the software snpEFF (snpEff_v2_0_5, http://snpeff.sourceforge.net/download.html) 30 and categorized into four classes (high, moderate, low and modifier), and the functional impact of coding variants was also predicted. Low‐impact variants were predicted by snpEFF and filtered out as they were synonymous coding and ‘assumed to be mostly harmless or unlikely to change protein behaviour’ as described in the manual (http://snpeff.sourceforge.net/SnpEff_manual.html). Sequence data were filtered against multiple databases using annovar (http://annovar.openbioinformatics.org, version 2013Aug23), and MAFs of the called variants were compared against dbSNP 137 (ftp://ftp.ncbi.nih.gov/snp/) and 1000 Genomes Project, where we filtered against the European cohort (EUR.MAF, April 2002 release, ftp://ftp.1000genomes.ebi.ac.uk/vol1/ftp). Then, variants were visualized with Integrative Genomics Viewer (IGV, version 2.3.36). The concordance between the genotypes of the variants identified through WES and bead SNP genotyping was evaluated using the PLINK program. Concordance was of 96% and 94% for UG and HC, respectively. A more detailed description can be found in the Supplementary Methods.

### Validation study: Sanger sequencing and variant segregation analysis

Segregating variants in affected individuals were prioritized based on their scaled C‐scores from the Combined Annotation Dependent Depletion (CADD) webserver (http://cadd.gs.washington.edu) 31. Priority variants were validated using Sanger sequencing on IgAN patients and controls from the WES group. Validated variants were further tested in extended families to evaluate their segregation in other pedigree members with urinary abnormalities. See Supplementary Methods for detailed information.

### Frequency evaluation: database query and TaqMan SNP genotyping

The frequencies of variants validated by Sanger sequencing were evaluated using the Exome Aggregation Consortium (ExAC; http://exac.broadinstitute.org) and the 1000 Genomes phase 3 (http://browser.1000genomes.org) databases. Genotyping was performed using TaqMan^®^ assays. Briefly, appropriate amplification primers were designed for the genomic region containing the variant of interest. TaqMan reactions were carried out in 25 μL volumes containing 10 ng DNA, according to the manufacturer's protocols. Amplification parameters were as follows: hot start at 95 °C for 10 min; 40 amplification cycles (95 °C for 15 s, 60 °C for 1 min). Results were obtained using a StepOnePlus Real‐Time PCR system and genotypes were called using stepone Software v2.2 (Life Technologies, Monza, Italy). Genotype clustering patterns were examined and confirmed to be of good quality. Assay controls with known genotype from Sanger sequencing were included in each plate.

### Network analysis to identify rare family variants belonging to known pathways

To assess biological relationships amongst genes, we used Ingenuity Pathway Analysis (IPA) software (Ingenuity Systems, Redwood City, CA, USA; http://www.ingenuity.com). We performed canonical pathway analysis. IPA generated networks based on the connectivity of the genes and computed a score for each network. These scores indicated the likelihood of focus genes belonging to a network versus those obtained by chance. A score of 2 indicates a 1 in 100 chance that the focus genes are together in a network because of random chance. The canonical pathway analysis identified the pathways from the IPA library of canonical pathways that were most significant to the input data set. Significance of the canonical pathway was calculated using Fisher's exact test. The whole data set composed of the 52 *in silico*‐identified segregating variants was not evaluated as it contained nonconfirmed variants or variants that were also present in the intrafamilial control. For network analysis, we uploaded 23 genes which contained 24 co‐segregating variants with the IgAN status. IPA associates a list of drug targets with generated networks based on knowledge base data (US Food and Drug Administration, Goodman and Gilman's The Pharmacological Basis of Therapeutics and Mosby's Drug Consult). The upstream regulator analysis has been used to identify a list of transcriptional regulators that explains observed gene expression changes in our data sets. The calculated overlap *P‐*value measures whether there is a statistically significant overlap between the dataset genes and the genes that are regulated by the transcription regulator. It is calculated using Fisher's exact test and significance is generally attributed to *P*‐values <0.01.

## Results

### GWLA of IgAN families

Sixteen multiplex families comprising 34 IgAN patients and 112 relatives were included in the linkage study (Figure S1). Nonparametric linkage ‘affected‐only’ analysis confirmed and refined the evidence for linkage to previously described susceptibility loci for IgAN on chromosomes 4q24–28 (LOD 2.4, *P* = 4.5 × 10^−4^), 6q22–23 (LOD 1.6, *P* = 3.2 × 10^−3^) and 17q12–21 (LOD 1.6, *P* = 3.3 × 10^−3^) 12, 13. Our previous linkage study was performed using 400 microsatellites 13; here, we adopted a SNP‐based strategy using a finer genetic map of 300 000 SNPs (see Supplementary material for detailed description). We found nine additional suggestive linked regions (LOD >1.5 or *P* < 5.0 × 10^−3^) in other areas of the genome (Table 2 and Figure S2). Interestingly, different families shared multiple linkage signals spread across 11 chromosomes. This prompted us to focus the sequencing on eight of these families that were providing the strongest contribution to multiple linkage signals in novel and known regions (Figure S3).

**Table 2:**
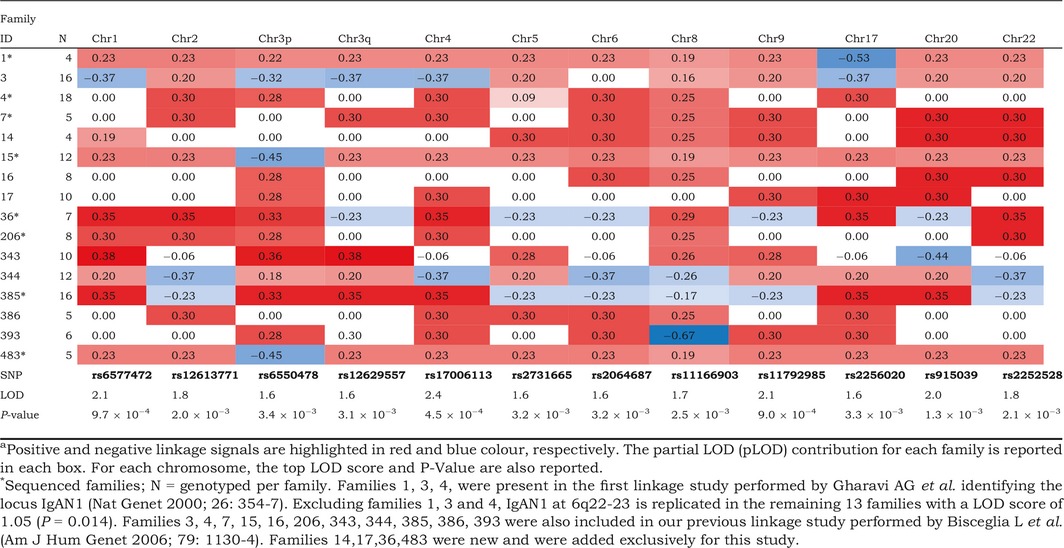
Sixteen families linked to each chromosomal region^a^

### WES of IgAN families

From each of the eight kindreds, we selected a trio comprising two affected individuals and an internal negative control showing in the regions of interest the least IBD sharing with the affected individuals 24, 25. This resulted in a total of 16 cases and eight familial controls to be sequenced (Figure S3).

We performed a target capture procedure able to target 62 Mb of the genome including the exome, microRNA sites and noncoding upstream and downstream regulatory regions. Parallel sequencing generated a mean of 3.6 billion bases of raw data per sample (range: 0.745–5.9 Gb). After mapping, 98% of reads mapped to the human reference genome with a mean coverage of 20×. Two distinct algorithms from the GATK were used to call variants: UG and HC (Table S1), identifying an average of 42 975 and 31 390 variants per individual, respectively.

The genetic variant annotation and the effect prediction toolbox SnpEff 30 were used to annotate variants based on their genomic locations, and the coding effects were also predicted.

**Figure 1:**
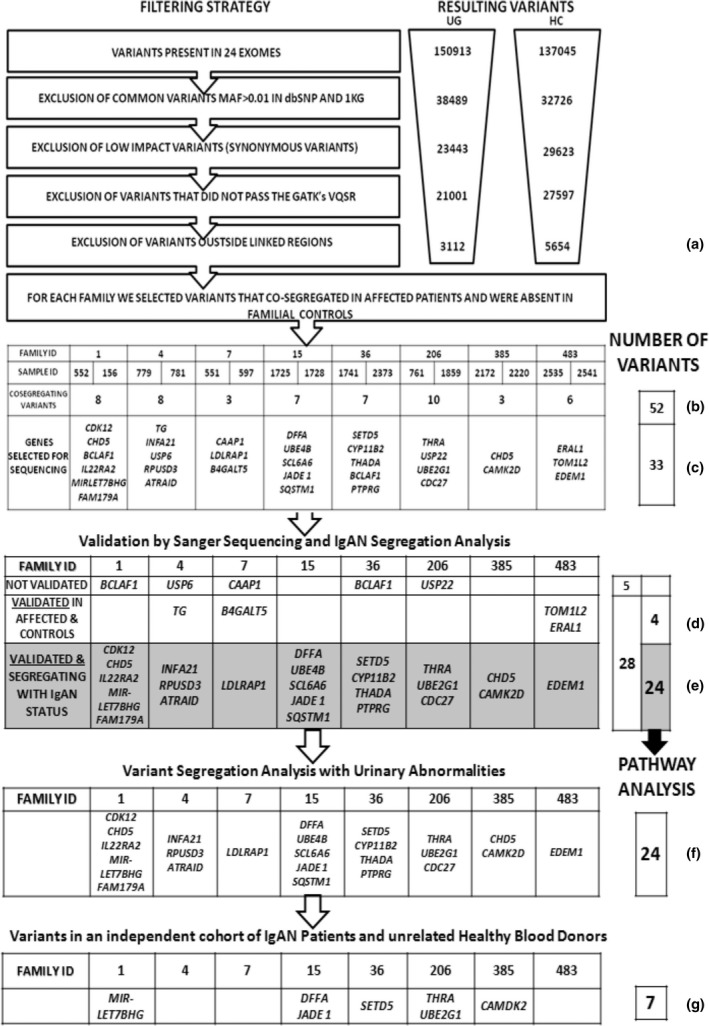
Overview of variant filtering and analysis strategy used to identify co‐segregating variants in affected patients with IgA nephropathy (IgAN). Two distinct algorithms from the Genome Analysis Toolkit (GATK) were used to call variants: Haplotype Caller (HC) and Unified Genotyper (UG). These algorithms generated 150 913 and 137 045 variants, respectively, in 24 exomes. Filtering was performed by removing (i) common variants defined by EUR.MAF >1%, (ii) low‐impact variants and (iii) variants that did not pass GATK filters. Linkage data were then included in the filtering step, and only variants located within linked genomic intervals were further considered (a). We identified 52 variants that co‐segregated in affected individuals and were absent in the intrafamilial control generating a set of co‐segregating variants (b). These variants were visually inspected using the Integrated Genomics Viewer, and 33 were selected for Sanger sequencing (c). Of these 33 selected variants, 28 were confirmed by Sanger sequencing. Four of these validated variants were also detected in unaffected controls (*B4GALT5, TOM1L2, ERAL1* and *TG*) (d). Thus, 24 variants segregated with the IgAN status and were used for pathway analysis (e). The same 24 validated variants were used to determine whether they were also co‐segregating with urinary abnormalities (f). Finally, we tested the frequency of seven randomly selected validated variants in an independent cohort of 240 IgAN patients and 113 unrelated healthy blood donors (g).

To identify susceptibility variants potentially involved in IgAN, we adopted a filtering strategy, illustrated in Fig. 1, based on (i) MAF (excluding common variants with EUR.MAF >0.01), (ii) ‘low’ effect prediction (excluding all synonymous coding SNPs) and (iii) quality of the variant based on GATK's Variant Call Quality Recalibration (VCQR). This approach led to a total of 21 001 and 27 597 called variants for UG and HC, respectively (Fig. 1). Some variants were called uniquely by one or the other algorithm so we decided to retain both UG and HC lists (Figure S4). Furthermore, we found that the vast majority of variants in each gene region were comparable between the two callers (Figure S5). Linkage data were then incorporated in the filtering pipeline and only variants located within linked genomic intervals (i.e. showing a LOD score >1.5 or *P* < 5 × 10^−3^) were considered further (Table S2), leading to the identification of 3112 and 5654 variants for UG and HC, respectively (Fig. 1a). Finally, priority was given to variants that co‐segregated in affected individuals and were absent in the intrafamilial control. This procedure was adopted to exclude co‐segregating variations due to the relatedness of the trios, thus highlighting the ones that were effectively involved with IgAN. Each set of co‐segregating variants per family was then visually inspected using the Integrated Genomics Viewer (Fig. 1b). Visual inspection permitted assessment of the variants in their genomic context, evaluation of repeat sequences and ascertainment that the variation was not an artefact. Furthermore, read pair orientation and overall depth of sequencing in cases and controls were evaluated. We decided to retain low coverage variants in the list of candidates to follow up only if detected in both affected individuals, as the likelihood of this occurring by chance is limited. Likewise, we decided to retain variants that were not contributing to the partial LOD in the linked region if co‐segregating, giving priority to the fact that variants were present in both affected individuals and absent in the intrafamilial control. This procedure identified 52 co‐segregating variants in affected individuals narrowing down to less than 1% of all variations called within the linked regions (Fig. 1b and Table S3).

Variants were annotated for scaled C‐scores using the unsupervised CADD score which prioritizes functional, deleterious and pathogenic variants across many functional categories. We found that none of the co‐segregating rare variants was present in more than one family. However, two genes, *BCLAF1* and *CHD5*, were found to contain distinct segregating variants in families 1, 36 and 385 (Fig. 1c).

### Validation study: Sanger sequencing and variant segregation analysis

We performed Sanger sequencing to validate the rare variants identified by WES and to confirm their segregation in affected individuals and their absence in the unaffected controls. Due to limited DNA availability for some of our families, we decided to validate the 33 most promising variants (Fig. 1c, Table 3 and Tables S3 and S4). Variant prioritization was performed in an unsupervised way based principally on CADD score (28 variants, score >5), gene expression in peripheral blood leucocytes of IgAN patients 32, 33, 34, 35 (*JADE1* 4:g129783008t>a, *UBE2G1* 17:g4173166g>a and *LDLRAP1* 1:g25894878c>g) and rare co‐segregating variants in the same gene in more than one family (*BCLAF1* 6:g136579552a>g and *BCLAF1* 6:g136579558a>g) (Table 3). Of the 33 variants, 28 (85%) were confirmed by direct sequencing (Fig. 1d,e) and the remaining five variants (*USP6 17:g5036210t>g, CAAP1 9:26841936c>a, USP22 17:g20931986g>t, BCLAF1 6:g136579552 and BCLAF1 6:g136579558*) were not present. The two variants predicted within the *BCLAF1* gene were excluded (see Figures S6 and S7). Four of the 28 variants (Fig. 1d) were validated by Sanger sequencing but also detected in the unaffected intrafamilial control (*B4GALT5* 20:g48250578t>c*, TOM1L2 17:g17748047g>a, ERAL1 17:g27188606a>c* and *TG 8:g133925492c>t*).

**Table 3:** Candidate gene variants selected for Sanger sequencing

Family ID	Gene symbol	Gene name	CHR	POS	REF	ALT	SnpEff effect	Sanger validation	ExAC	1000G phase 3
1	*CHD5*	Chromodomain helicase DNA binding protein 5	1	6 163 696	G	A	DOWNSTREAM	Yes	NC	Absent
1	*FAM179A*	Family with sequence similarity 179, member A	2	29 249 757	AC	A	FRAME_SHIFT	Yes	Absent	Absent
1	*IL22RA2*	Interleukin 22 receptor, alpha 2	6	137 465 358	C	T	UTR_3_PRIME	Yes	NC	Absent
1	*CDK12*	Cyclin‐dependent kinase 12	17	37 689 446	C	T	UTR_3_PRIME	Yes	NC	0.003
1	*MIRLET7BHG*	MIRLET7B host gene	22	46 453 973	T	C	INTRON	Yes	NC	Absent
4	*ATRAID*	All‐trans retinoic acid‐induced differentiation factor	2	27 439 820	A	G	DOWNSTREAM	Yes	NC	Absent
4	*RPUSD3*	RNA pseudouridylate synthase domain containing 3	3	9 880 772	T	C	NON_SYNONYMOUS_CODING=T255A	Yes	0.00015	Absent
4	*IFNA21*	Interferon, alpha 21	9	21 165 905	C	T	UTR_3_PRIME	Yes	NC	Absent
7	*LDLRAP1*	Low‐density lipoprotein receptor adaptor protein 1	1	25 894 878	C	G	DOWNSTREAM	Yes	NC	0.0001
15	*UBE4B*	Ubiquitination factor E4B	1	10 190 827	C	T	NON_SYNONYMOUS_CODING=R378C	Yes	0.000016	Absent
15	*DFFA*	DNA fragmentation factor, 45 kDa, alpha polypeptide	1	10 527 277	G	C	NON_SYNONYMOUS_CODING=S137R	Yes	0.0000082	Absent
15	*SLC6A6*	Solute carrier family 6 (neurotransmitter transporter), member 6	3	14 528 787	A	G	UTR_3_PRIME	Yes	NC	0.00059
15	*JADE1*	Jade family PHD finger 1	4	129 783 008	T	A	NON_SYNONYMOUS_CODING=S365R	Yes	0.00001648	Absent
15	*SQSTM1*	Sequestosome 1	5	179 264 117	A	G	DOWNSTREAM	Yes	NC	Absent
36	*THADA*	Thyroid adenoma associated	2	43 455 302	G	A	DOWNSTREAM	Yes	NC	Absent
36	*SETD5*	SET domain containing 5	3	9 515 095	C	A	NON_SYNONYMOUS_CODING=S1026Y	Yes	Absent	Absent
36	*PTPRG*	Protein tyrosine phosphatase, receptor type, G	3	62 063 912	G	A	NON_SYNONYMOUS_CODING=A199T	Yes	0.000008341	Absent
36	*CYP11B2*	Cytochrome P450, family 11, subfamily B, polypeptide 2	8	143 993 975	C	T	NON_SYNONYMOUS_CODING=E457K	Yes	Absent	Absent
206	*THRA*	Thyroid hormone receptor, alpha	17	38 233 146	C	T	STOP_GAINED=R26*	Yes	Absent	Absent
206	*UBE2G1*	Ubiquitin‐conjugating enzyme E2G 1	17	4173 166	G	A	UTR_3_PRIME	Yes	NC	Absent
206	*CDC27*	Cell division cycle 27	17	45 197 967	A	G	DOWNSTREAM	Yes	NC	0.000199681
385	*CAMK2D*	Calcium/calmodulin‐dependent protein kinase II delta	4	114 374 628	T	A	DOWNSTREAM	Yes	NC	0.00259585
385	*CHD5*	Chromodomain helicase DNA binding protein 5	1	6 162 250	G	GAC	DOWNSTREAM	Yes	NC	0.0071885
483	*EDEM1*	ER degradation enhancer, mannosidase alpha‐like 1	3	5 259 973	A	G	UTR_3_PRIME	Yes	NC	Absent
4	*TG*	Thyroglobulin	8	133 925 492	C	T	STOP_GAINED=Q1454*	Yes + Control	0.000008248	Absent
7	*B4GALT5*	UDP‐Gal:betaGlcNAc beta 1,4‐ galactosyltransferase, polypeptide 5	20	48 250 578	T	C	UTR_3_PRIME	Yes + Control	NC	Absent
483	*TOM1L2*	Target of myb1‐like 2 (chicken)	17	17 748 047	G	A	DOWNSTREAM	Yes + Control	NC	0.000599042
483	*ERAL1/MIR144/*	Era‐like 12S mitochondrial rRNA chaperone 1	17	27 188 606	A	C	DOWNSTREAM	Yes + Control	NC	Absent
1	*BCLAF1*	BCL2‐associated transcription factor 1	6	136 579 552	A	G	DOWNSTREAM	Repeat region	NC	Absent
36	*BCLAF1*	BCL2‐associated transcription factor 1	6	136 579 558	A	G	DOWNSTREAM	Repeat region	NC	Absent
4	*USP6*	USP6 ubiquitin specific peptidase 6	17	5 036 210	T	G	NON_SYNONYMOUS_CODING=I67M	Not confirmed	NC	Absent
7	*CAAP1*	Caspase activity and apoptosis inhibitor 1	9	26 841 936	C	A	DOWNSTREAM	Not confirmed	NC	Absent
36	*USP22*	Ubiquitin specific peptidase 22	17	20 931 986	G	T	NON_SYNONYMOUS_CODING=A126D	Not confirmed	NC	Absent

Chr, chromosome; Pos, position; Ref, reference allele; Alt, alternate allele; SnpEff EFFECT, prediction of variant effect on gene based on SnpEff software (http://snpeff.sourceforge.net/download.html); ExAC, Exome Aggregation Consortium variant frequency data (http://exac.broadinstitute.org); NC, no variant detected because not in coding regions; Absent, no variant detected. 1000G phase 3, variant frequency data from the 1000 Genomes phase 3 database (http://browser.1000genomes.org).

On the whole, WES data were confirmed with Sanger sequencing on 28 variants of which 24 were found to co‐segregate with the IgAN affection status. Most of these variants (63%, 15/24) fell within untranslated or noncoding regulatory regions of the genome.

**Figure 2:**
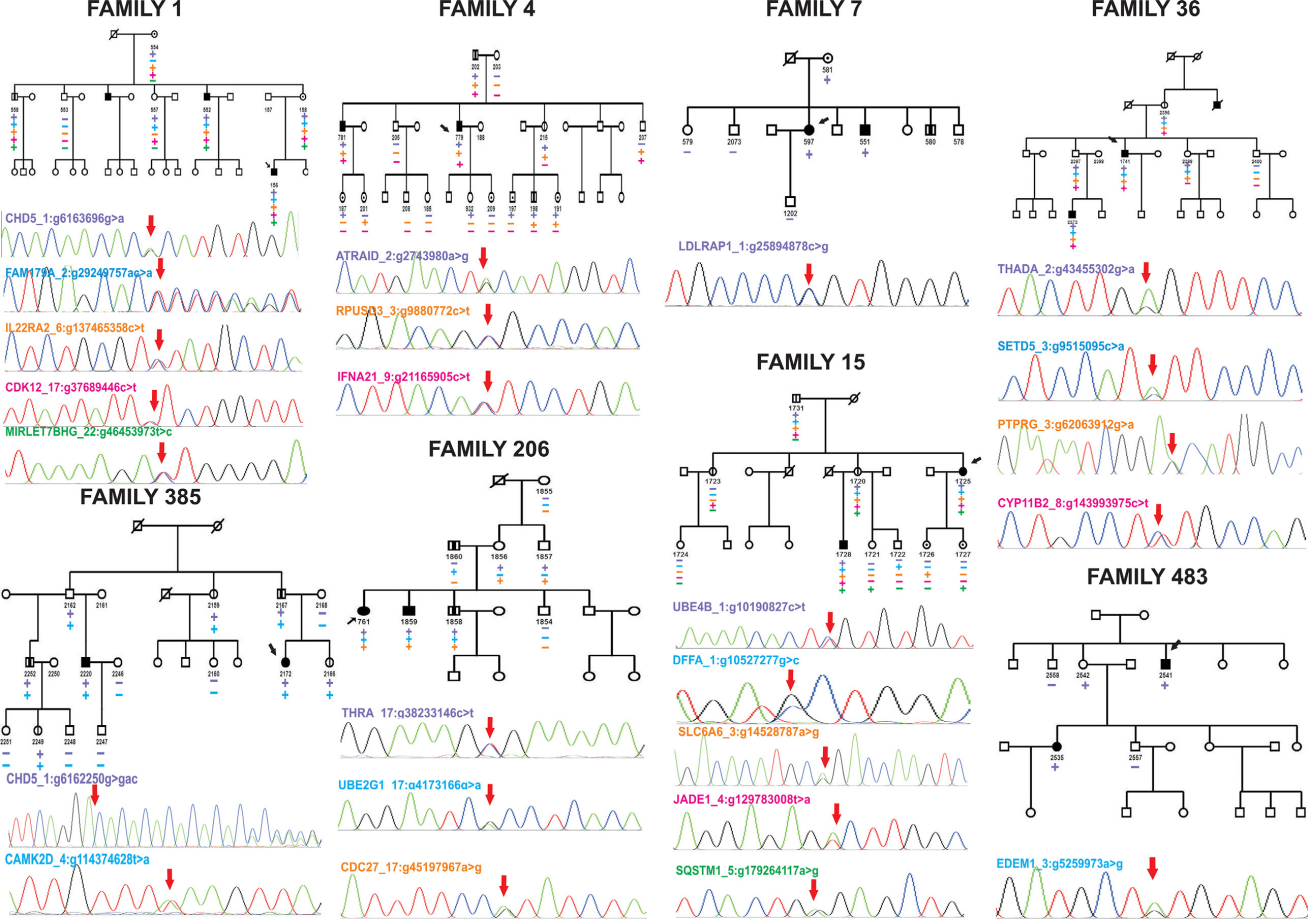
Rare variant segregation patterns in families with IgA nephropathy (IgAN). Heterozygous rare mutations that segregated with the affection status were evaluated in extended family members. Squares and circles represent males and females, respectively; arrows indicate probands, and slashes indicate deceased individuals. Filled and unfilled symbols indicate individuals affected and unaffected by IgAN, respectively. Symbols with a dot indicate individuals with an unknown phenotype (individuals without urinalysis or who had intermittent microscopic haematuria). Symbols with a vertical line indicate individuals with documented urinary abnormalities (persistent microscopic haematuria and/or proteinuria). The plus symbol indicates individuals who carry the corresponding heterozygous mutation, and the minus symbol indicates those who carry the wild‐type allele at this locus. The panels under each pedigree show representative mutant electropherograms of the sequencing products obtained in the index patient (red arrow).

We investigated whether all the 24 variants that were segregating with biopsy‐proven IgAN status were also segregating with urinary abnormalities, that are persistent microscopic haematuria and/or mild proteinuria, in extended family members included in WES (Figs 1f and 2, and Table S4). A complete description of the variant segregation in extended families can be found in the Supporting information. Overall, all tested variants segregated both with the IgAN status and with at least one other family member with urinary abnormalities. Moreover, in some families we detected putative obligate carriers. These subjects were characterized by normal urinalysis but carried all the mutations (2397, 2162 and 2542); the presence of putative obligate carriers within pedigrees is consistent with the autosomal dominant mode of transmission of this disease with incomplete penetrance for IgAN 13, 14, 15, 36 and suggests that environmental triggers together with genetic factors are needed for disease onset 15, 37. The identification of multiple mutations in affected individuals suggests that each one may cumulatively concur in determining the disease.

### Frequency evaluation: database query and TaqMan SNP genotyping

Next, we compared the frequencies of all 28 Sanger sequencing‐validated variants using two different databases, the ExAC and the 1000 Genomes phase 3 databases; the former is the largest publicly accessible exome‐sequencing data set reporting population frequency data of 60 706 unrelated individuals and the latter contains genomewide genotype data of 2504 individuals. We found that amongst the 28 validated variants, 15 were private and the remaining 13 were present in the databases with a low frequency (MAF <0.003) (Table 3). Then, we tested whether the frequencies of seven randomly selected variants were higher in an independent cohort of 240 IgAN patients compared with 113 unrelated HBDs (Fig. 1g). Furthermore, we also tested all families who did not undergo WES, but contributed to the linkage peaks from the original GWLA study. The custom TaqMan SNP genotyping assays were successfully designed for the following variants: *CAMK2D* 4:g114374628t>a, *THRA* 17:g38233146c>t, *MIRLET7BHG* 22:g46423973t>c, *JADE1* 4:g129783008t>a, *DFFA* 1:g10527277g>c, *SETD5* 3:g9515095c>a and *UBE2G1* 17:g4173166 g>a (Table S5). For each variant tested, we found that it was only detected in the WES cohort in which it was first discovered. No other IgAN patients or HBDs carried the tested mutations. These results suggest that the tested variants are rare with a frequency of <0.002 in our IgAN population.

### Network analysis to identify rare family variants belonging to known pathways

**Figure 3:**
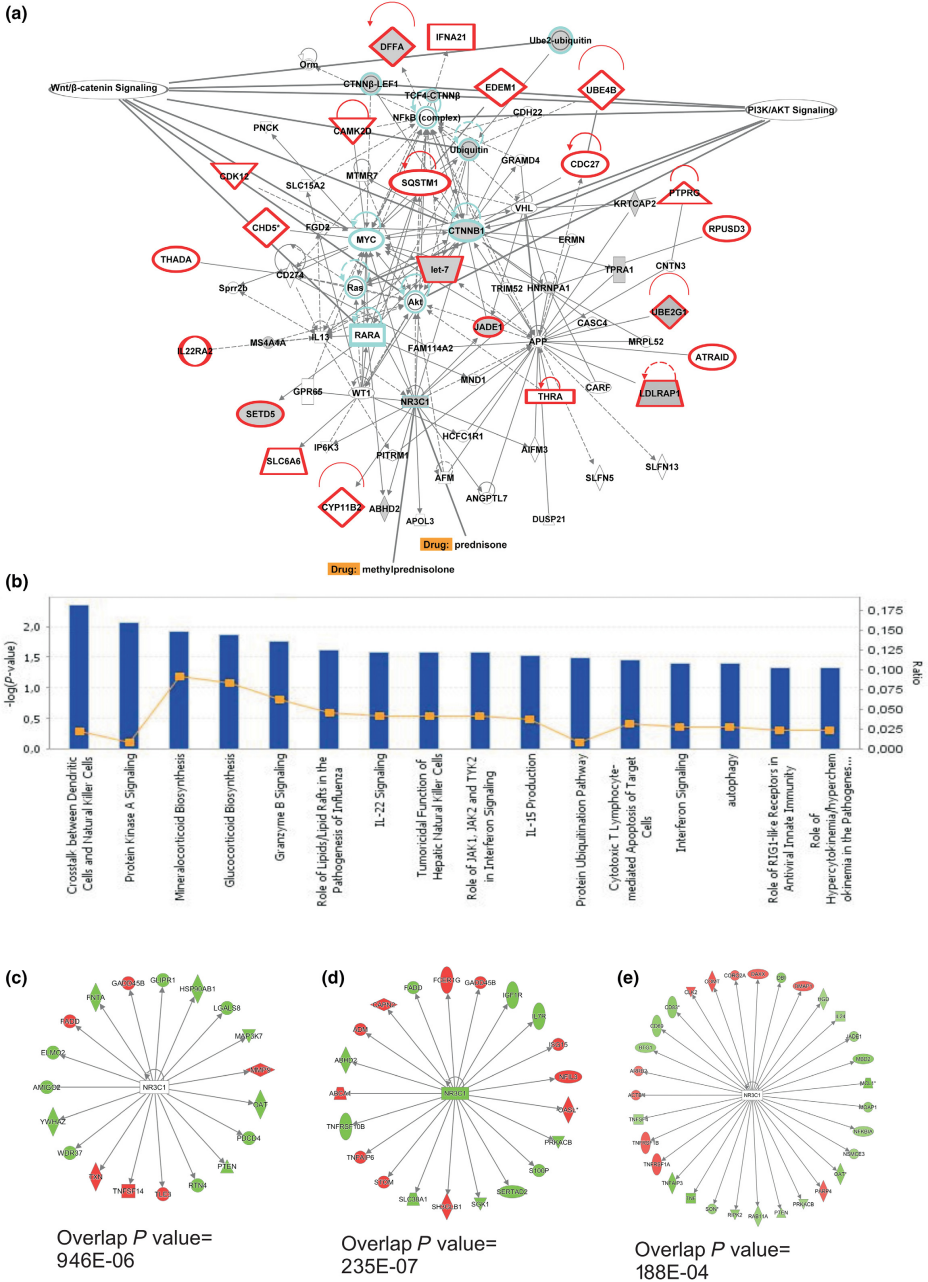
Functional analysis of the network containing validated segregating variants. The network was algorithmically constructed using Ingenuity Pathway Analysis (IPA) software based on the functional and biological connectivity of genes (a). The network is graphically represented as nodes (genes) and edges (the biological relationship between genes). Red nodes represent genes containing identified variants; others (empty nodes) are those that IPA automatically includes because they are biologically linked to the studied genes based on evidence in the literature. This is the only network that was generated by the software (score 59, *n =* 22 associated genes). *UBC*,*AKT*,*CTNNB1* and *NFKB* are central nodes computed by the software (cyan colour). We overlaid onto this network previously published expression data obtained from IgAN patients and found that differently expressed genes and complexes were included in this network (*ABHD2, CTNNB1, CTNN*β*‐LEF1, DFFA, HNRNPA1, JADE1, KRTCAP2, LDLRAP1, let‐7, MS4A4A, NR3C1, SETD5, TCF4‐CTNN*β*, TPRA1, Ube2‐ubiquitin, UBE2G1 and Ubiquitin*; grey shaded symbols). The most representative canonical pathways associated with the uploaded genes are mainly involved in innate and adaptive immunity (b). The glucocorticoid receptor gene (*NR3C1*) is the target of steroid therapy (prednisone and methylprednisolone) routinely used in IgAN treatment (a). The upstream regulator analysis identified *NR3C1* as a transcriptional regulator that explains the observed gene expression changes in differently expressed genes in peripheral blood cells (c, *P* = 9.46 × 10^−6^), during the acute phase of the disease (d, *P* = 2.03 × 10^−7^) and in monocytes isolated from IgAN patients (e, *P* = 1.88 × 10^−4^).

We investigated whether the 24 variants that co‐segregate with the IgAN affection status within 23 apparently unconnected genes were functionally related and concurred to modulate a common pathway. We tested this hypothesis *in silico* using IPA Knowledge Base. This unsupervised analysis identified a single network characterized by a high connectivity score of 59 (Fig. 3a). Of 23 uploaded genes, 22 were within this network (Fig. 3a, red shaded genes). We found that *AKT, CTNNB1, NFKB, MYC and UBC* were included as central hubs of the network and all belonged to WNT/β‐catenin and PI3K/Akt signalling (Fig. 3a, cyan coloured symbols). All these genes and pathways are implicated in disease pathogenesis and are aberrantly expressed both at gene and protein levels in IgAN patients 35, 38, 39, 40. In particular, when we overlaid our previously published expression data onto this network, we found that genes and complexes with altered expression in IgAN were also included in this network (*ABHD2, CTNNB1, CTNN*β*‐LEF1, DFFA, HNRNPA1, JADE1, KRTCAP2, LDLRAP1, let‐7, MS4A4A, NR3C1, SETD5, TCF4‐CTNN*β*, TPRA1, Ube2‐ubiquitin, UBE2G1* and *Ubiquitin* (Fig. 3a, grey shaded symbols) 32, 33, 35, 40, 41. The representative canonical pathways associated with the uploaded genes are mainly involved in innate and adaptive immunity (Fig. 3b).

Notably, the network included the gene encoding the glucocorticoid receptor, also known as *NR3C1,* which is the target of steroid therapy routinely used in the treatment of IgAN. Corticosteroids are able to lower the risk of kidney failure and reduce proteinuria 2, 42, 43 (Fig. 3B). Furthermore, all gene expression studies performed on IgAN patients showed that the receptor *NR3C1* significantly influences various downstream molecules aberrantly expressed in these patients 33, 34, 35 (Fig. 3c–e: *P* = 9.46 × 10^−6^, *P* = 2.03 × 10^−7^ and *P* = 1.88 × 10^−4^, respectively). The complete list of potential drugs that act on this specific IgAN‐related network is shown in Table S6.

In conclusion, our results suggest that IgAN‐segregating variants interact with each other generating a single network in which altered immune‐related pathways have a central role. This network could highlight potential targets for a targeted and personalized therapy.

## Discussion

This is the first study designed to identify rare, highly penetrant risk variants involved in IgAN integrating two different genomewide approaches, GWLA and WES. We identified 24 variants segregating with IgAN patients and absent in their intrafamilial controls. Furthermore, we confirmed that these variants were either rare or private and were characterized by a strong functional interconnectivity. In addition, these variants appeared in a single network connected by central hubs highly relevant to IgAN.

We first performed a GWLA using a finer SNP map compared with previous linkage studies carried out by microsatellites. The cumulative LOD scores generated supported the previous linkage signals on chromosome 6q22–23 12 and on chromosomes 4q26–31 and 17q12–22 13. The identification of additional linked loci is consistent with a multiple susceptibility gene model for familial IgAN, as suggested in previous linkage studies in IgAN 12, 13, 14. Furthermore, these linked loci were once again distinct from the genomic areas identified by GWASs 5, 6, 7, 8, 9. This discrepancy may be ascribed to (i) differences in the genetic approach, the former identified through a family‐based approach and the latter using population‐based approaches 44, and (ii) potential underlying differences in the genetic determinants in familial and sporadic IgAN 45.

GWLA was guidance for next‐generation sequencing, and the results were used to provide an informative, strictly selected subset of familial cases enriched with putative highly penetrant genetic risk variants, and genetically discordant intrafamilial controls to be sequenced. This approach was adopted to minimize the impact of population stratification, as the quality control methods developed for GWASs of common variants should also be applied to rare variant studies 46.

After applying filtering criteria and having visually inspected the genomic context, we identified 52 co‐segregating variants. They were selected due to their presence in both affected individuals and absence in unaffected intrafamilial sequencing controls. Because the number of rare variants in the human genome is very high 47, this approach excluded co‐segregating variants due to the relatedness of the trios and rapidly narrowed down the potentially causative and co‐segregating variants. The segregation analysis in our extended families demonstrated that variants segregated with the IgAN status and in individuals with persistent microscopic haematuria and/or mild proteinuria. Furthermore, we also detected putative obligate carriers in unaffected family members. The presence of putative obligate carriers within pedigrees is consistent with the autosomal dominant mode of transmission of this disease with incomplete penetrance for IgAN 13, 14, 15, 36. These obligate carriers provide evidence that IgAN may be determined by multiple genes and environmental factors that interact in a complex network 15, 37. A multihit model has been postulated for IgAN 11, 48 in which other environmental factors are needed for disease onset, with genetic risk having recently been shown to correlate strongly with variation in local pathogens. In particular, helminth diversity confers an increased risk in IgAN 8.

Then, we sought to evaluate whether the variants identified in our WES cohort were also present in other well‐characterized IgAN patients and unrelated HBDs, but none carried the tested variants. The failure to identify a single rare segregating variant in more than one family or any of the rare variants in the other tested familial or sporadic IgAN cases supports the hypothesis that this complex and fairly common disease may be ascribed not to a single gene or variant but to a cumulative effect of rare variants 49.

Finally, we focused on the 24 validated variants within 23 genes that fulfilled the segregation criteria in affected individuals and were not carried by unaffected controls. The genes were interconnected in a single functional network with *AKT, CTNNB1, NFKB, MYC* and *UBC* as central nodes. These genes appear to be biologically relevant because they are key modulators of WNT/β‐catenin and PI3K/Akt pathways, which are altered in this disease 35, 39, 40, 41, 50.

The network included the glucocorticoid receptor gene, *NR3C1*, which is the target of corticosteroid therapy recommended by the KDIGO guidelines for the treatment of IgAN 2, 43, 51. We found that *NR3C1* significantly influences various downstream molecules aberrantly expressed in IgAN patients and its modulation may contribute to the beneficial effects of NR3C1 agonists in this disease 2, 42, 43. Due to the increased risk of adverse events, the use of corticosteroids in IgAN has been recently debated 52, 53, 54, 55 and evokes the need for a more targeted therapy. The analysis of other drug targets included in this IgAN‐related network could be a starting point for future studies on personalized therapies (Table S6).

Recently, a WES study has been performed on individuals of Chinese Han ethnicity with familial IgAN 56 and seven very common co‐segregating deleterious variants within five genes have been identified. These variants were not present in our filtered data set as we applied the stringent filtering criteria of MAF <1%. We further checked our data and found that there were no rare segregating variants within these genes. This difference could also be ascribed to the different ethnicity.

Our study has several limitations. First, because of the limited availability of DNA, it is not possible to validate all the segregating variants identified bioinformatically. Nevertheless, we pursued 64% of the variants (33 of 52) based mainly on the systematic selection of their higher CADD score, which is able to prioritize variants across functional categories and effect sizes. Secondly, the effect of each variation was not evaluated experimentally. As an alternative approach, we overlaid our previously validated experimental gene and protein expression data onto the functional network generated by IPA and found that statistically significant differently expressed genes/complexes in IgAN were included in the network. Further studies are needed to understand whether these variants can effectively disrupt normal function of their relevant genes and whether such a disruption may contribute to our phenotype of interest.

Despite these limitations, our study has several strengths. First, our results suggest a multiple susceptibility gene model for familial IgAN. Secondly, we have identified many rare segregating variants that are involved in a common pathway mostly with a role in innate and adaptive immunity. Thirdly, the identified network contains genes and pathways previously demonstrated to be dysregulated in IgAN and drug targets that could be exploited in the treatment of the disease.

IgAN is characterized by an altered innate immunity 50 with a hyper‐responsiveness of the IgA immune system and an increased IgA‐secreting plasma cell number in both bone marrow and tonsils 57, 58. These cells show a reduced susceptibility to Fas‐mediated apoptosis with marked expression of bcl‐2 59. Furthermore, WNT/β‐catenin and PI3K/Akt pathways are hyperactivated in peripheral blood mononuclear cells with increased Akt phosphorylation, β‐catenin and NFkB nuclear translocation 35, 39 ultimately leading to an enhanced cell proliferation rate. The uncovering of 23 variant‐containing genes, strictly connected to these aberrant networks, may in part explain the IgAN cell hyperactivated phenotype, thus partly revealing the pathogenic mechanisms of IgAN.

## Conclusions

Together, our results support the novel hypothesis that IgAN is caused by distinct rare segregating variants, which may act within a specific IgAN immune‐related network. Our findings could be used in future studies to test novel drug targets for the treatment of this disease.

## Conflict of interest

The authors declare no conflicts of interest.

## Availability of data and materials

Microarray data of 146 IgAN patients and relatives genotyped on Illumina HumanCytoSNP‐12 have been previously deposited in NCBI's Gene Expression Omnibus (GEO) database under the accession number GSE44974. WES data for all families have been deposited in NCBI's SRA database (http://www.ncbi.nlm.nih.gov/sra) and are available under the study accession number SRP061415.

## Funding

This work was supported by grants from the European Framework Programme (QLG1‐CT‐2000‐00464, to FPS), MRC (MR/K01353X/1, to MF), MiUR (PON‐REC ONEV 134/2011 and FIRB RBAP11B2SX to FPS), Regione Puglia (BISIMANE project, 44/2008 to FPS) and the Ministry of Health (GR‐2011‐02350438 to GS). FP was supported by long‐term fellowships from the European Renal Association–European Dialysis and Transplant Association (ERA‐EDTA ALTF 72‐2010 and ERA‐EDTA ALTF 89‐2011). We are grateful to the Schena Foundation for scientific and financial support.

## Supporting information


**Figure S1.** Sixteen multiplex families included in the linkage study, the red bars represent 146 genotyped subjects.
**Figure S2**. Plot of LOD score statistics from NPL analysis for the chromosomes in which the score exceeded the 1.5 level.
**Figure S3.** Pedigrees included in the exome sequencing study.
**Figure S4.** Number of variants called by Unified Genotyper (UG) and Haplotype Caller (HC).
**Figure S5.** Percentage distributions of gene variantions as depicted in the output of SnpEff: Variant analysis.
**Figure S6.** The two variants predicted within the BCLAF1 gene were excluded as they were actually determined by the presence of a deletion in a repeated (AAAAAC)n region (and not by a G/A substitution).
**Figure S7.** We evaluated BCLAF1 complex genomic region in six unrelated HBD
**Table S1.** Summary of the exome sequencing results.
**Table S2.** Genomic intervals considered for variant filtration.
**Table S3.** List of co‐segregating gene variants identified by exome sequencing.
**Table S4.** Left and right PCR primers designed to amplify the genomic region containing the variant of interest used for Sanger validation.
**Table S5.** List of primers designed for SNP genotyping analysis using custom TaqMan assays.
**Table S6.** List of potential drugs targeting the network.
