# Supplementary material for: Multiple rare genetic variants co‐segregating with familial IgA nephropathy all act within a single immune‐related network
Source: J Intern Med. 2016 Oct 11;281(2):189–205. doi: 10.1111/joim.12565 (PMC5297991; doi:10.1111/joim.12565)
Supplement: Supplementary file 1 — Figure S1. Sixteen multiplex families included in the linkage study, the red bars represent 146 genotyped subjects. Figure S2. Plot of LOD score statistics from NPL analysis for the chromosomes in which the score exceeded the 1.5 level. Figure S3. Pedigrees included in the exome sequencing study. Figure S4. Number of variants called by Unified Genotyper (UG) and Haplotype Caller (HC). Figure S5. Percentage distributions of gene variantions as depicted in the output of SnpEff: Variant analysis. Figure S6. The two variants predicted within the BCLAF1 gene were excluded as they were actually determined by the presence of a deletion in a repeated (AAAAAC)n region (and not by a G/A substitution). Figure S7. We evaluated BCLAF1 complex genomic region in six unrelated HBD Table S1. Summary of the exome sequencing results. Table S2. Genomic intervals considered for variant filtration. Table S3. List of co‐segregating gene variants identified by exome sequencing. Table S4. Left and right PCR primers designed to amplify the genomic region containing the variant of interest used for Sanger validation. Table S5. List of primers designed for SNP genotyping analysis using custom TaqMan assays. Table S6. List of potential drugs targeting the network. [file JOIM-281-189-s001.pdf]

Supplementary online materials

Multiple Rare Genetic Variants Co-Segregating with Familial IgA Nephropathy all Convey into a Single Immune-Related Network

Sharon Natasha Cox,[1,2$&], Francesco Pesce,[1,3$], Julia Sarah El-Sayed Moustafa,[3,4], Fabio Sallustio[1], Grazia Serino,[1,5] Charalambos Kkoufou,[3], Annalisa Giampetruzzi,[6], Nicola Ancona,[7], Mario Falchi,[3,4*], and Francesco Paolo Schena,[1,2*], on behalf of the European IgAN Consortium.

[1]Dept of Emergency and Organ Transplantation. University of Bari Aldo Moro, Bari, Italy.

[2]C.A.R.S.O. Consortium, University of Bari, Italy;

[3]Dept of Genomics of Common Disease, Imperial College London, London, UK.

[4]Dept of Twin Research and Genetic Epidemiology, King's College London, London, UK.

[5]IRCCS "de Bellis", Laboratory of Experimental Immunopathology, 70013 Castellana Grotte, Bari, Italy.

[6] Dept of Soil, Plant and Food Sciences, University of Bari Aldo Moro, Bari, Italy.

[7]ISSIA, CNR, Bari, Italy,

[$]These authors contributed equally
*Francesco Paolo Schena and Mario Falchi shared senior authorship
[&]Corresponding Author

Running Title: Rare Genetic Variants in IgA Nephropathy

Key Words: KIDNEY DISEASE, FAMILY MEDICINE, GENE POLYMORPHISM, GENETICS, GLOMERULONEPHRITIS.

Correspondence: Cox Sharon Natasha, C.A.R.S.O. Consortium, University of Bari, 70100 Valenzano (Ba), Italy. Phone: 0039/080-4602121; FAX: 0039/080-4602121; e-mail: sharonnatasha.cox@uniba.it

DESCRIPTION OF SUPPLEMENTAL DATA:

Supplementary Text: Previous linkage study by Bisceglia *et al* as compared to this one. Analysis of variant segregation in extended families

Supplementary Methods: Sample donors, Microarray Genotyping, whole-exome sequencing, variant selection and Sanger Sequencing .

REFERENCES

Supplementary Figures S1-S7

Supplementary Tables S1-S6.

Supplementary text:

Previous linkage study by Bisceglia et al as compared to this one

The previous linkage studies by Gharavi AG et al [1] and Bisceglia et al [2]) were performed using 400 microsatellites, here we adopted a SNP based strategy using a finer genetic map of 300,000 SNPs. Between studies we have a partial overlap. Families 1, 3, 4, were included in the first linkage study perfomed by Gharavi AG et al (Nat Genet 2000; 26: 354-7). Families 3, 4, 7, 15, 16, 206, 343, 344, 385, 386, 393 were also present in our previous linkage study perfomed by Bisceglia L et al (Am J Hum Genet 2006; 79: 1130-4). The families 14,17,36,483 were added exclusively for this study. Compared to the previous papers, some subjects from each family have been added for genotyping.

Analysis of variant segregation in extended families

We investigated whether variants identified and validated in the previous steps were also co-segregating with urinary abnormalities, i.e. persistent microscopic hematuria and/or proteinuria, in unaffected family members of the extended families used for the WES.

In family 1, all validated variants (*CHD5* 1:g6163696g>a; *FAM179A* 2:g29249757ac>a; *IL22RA2* 6:g137465358c>t; *CDK12* 17:g37689446c>t; *MIRLET7BHG* 22:g46453973t>c) segregated in a family member who had microscopic hematuria (558) and was absent in those displaying negative urinalysis (553).

In Family 4 we found that out of the three validated variants (*IFNA21* 9:g21165905c>t; *ATRAID* 2:g27439820a>g; *RPUSD3* 3:g9880772t>c). *ATRAID* and *RPUSD3* segregated perfectly family members with IgAN and with persistent microscopic hematuria in family members 215, 202, 198. The *IFNA21* variation, on the other hand, was present in only one of the family members with microscopic hematuria (202).

In family 7, we evaluated the segregation of the variant 1:g25894878c>g within the *LDLRAP1* gene. This variant was absent in family members that had normal urinalysis (579, 2073, 1202) and was carried by a family member who presented some episodes of microscopic hematuria (581).

In family 15, five validated variants (*DFFA* 1:g10527277g>c; *JADE1* 4:g129783008t>a; *SLC6A6* 3:g14528787a>g; *SQSTM1* 5:g179264117a>g; *UBE4B* 1:g10190827c>t) segregated in subjects 1731 and 1720, both characterized by persistent microscopic hematuria and/or proteinuria. Only the non-synonymous coding variant S365R *JADE1* segregated in all three subjects characterized (1723, 1731 and 1720) by urinary abnormalities.

In family 36, all tested variants (*CYP11B2* 8:g143993975c>t, *SETD5* 3:g9515095c>a, *PTPRG* 3:g62063912g>a, *THADA* 2:g43455302g>a) segregated in two obligate carriers (2396 and 2397) and in a family member characterized by episodes of microscopic hematuria.

For family 206, the variants *THRA* 17:g38233146c>t, *UBE2G1* 17:g4173166g>a and *CDC27* 17:g45197967a>g that segregated with the affection status also segregated with an individual characterized by persistent microscopic hematuria (1858), while only *UBE2G1* gene variant segregated with a second individual characterized by persistent microscopic hematuria.

In family 385, the *CAMKD2* 4:g114374628t>a and the *CHD5* 1:g6162250g>gac variant was evaluated for segregation in the extended family. This variant was present in family members with persistent microscopic hematuria and/or proteinuria and in the subject 2162, an obligate carrier with a normal phenotype.

For family 483, we evaluated the co segregating variant 3:g5259973a>g within the *EDEM1* gene. This variant was absent in family members who had normal urinalysis apart from the obligate carrier 2542 with normal urinalysis.

Supplementary methods:

Sample donors

The genome-wide linkage analysis involved 34 biopsy-proven familial IgAN patients and 112 relatives from 16 Italian kindreds of South Italian ancestry (Table 1, Figure S1). Recruitment strategies and criteria for diagnosis have been reported elsewhere and are available on the European IgAN Consortium Web site (www.igan.net)[3]. Briefly , familial IgAN was diagnosed when at least 2 family members had biopsy-proven IgAN, the remaining family members were all checked for urinalysis. Some family members were affected by persistent microscopic hematuria and/or proteinuria and were depicted with an unknown status. Unaffected family members had at least three documented negative urinalysis. An independent cohort of 240 biopsy-proven IgAN patients and 113 HBD were included in the study for custom TaqMan SNP genotyping assays. Written informed consent was obtained from all study participants. The study was carried out according to

the principles of the Declaration of Helsinki and was approved by the local Institutional Ethics Review Board.

Microarray Genotyping

DNA was isolated from whole blood of IgAN patients and HBD by Qiagen QIAamp DNA Blood Midi Kit (Qiagen Srl, Milan, Italy). DNA was quality-checked on agarose gels and quantified using a microvolume spectrophotometer (NanoDrop 2000; Thermo Fisher Scientific Inc.). Genotyping was performed using Illumina HumanCytoSNP-12 BeadChip containing 300,000 selected tag SNPs with a median marker spacing of 6.2 kbases. Data was exported from Genome Studio Software. Microarray data of the IgAN patients and relatives genotyped on Illumina HumanCytoSNP-12 have been previously published by our group under accession number GSE44974 at the GEO (http://www.ncbi.nlm.nih.gov/geo/)[4].

Whole-exome sequencing

We performed whole exome sequencing on 16 most informative IgAN patients belonging to 8 non-consanguineous families and 8 intra-familial controls. For the selection of the internal (intra-family) negative controls, we performed an Identical By Descent (IBD) analysis on each of these 8 families and identified the closest relative (for each affected) with the least IBD-sharing (genetically discordant) in the region of interest.[5, 6] Three micrograms (μg) of genomic DNA was used for generating each exome library. Genomic DNA was randomly sheared using a Nebulizer (Life Technologies), adapters were ligated to each end of the fragments and purified using a QIAquick PCR Purification kit (Qiagen). Target regions were captured with the TruSeq Exome Enrichment and the illumina HiScanSQ system was used for sequencing. The magnitude of enrichment of captured ligation-mediated PCR products was determined using the Agilent 2100 Bioanalyzer. Next, each captured library was loaded onto the HiScanSQ platform, and paired-end sequencing was performed with read lengths of 101bp. Image analysis was performed with default parameters of Illumina RTA v1.13 pipeline and demultiplexing was performed with CASAVA 1.8.2 (Illumina). Sequence reads were mapped to the reference human genome (UCSC Genome Browser hg19) using the Burrows-Wheeler aligner (BWA; version 0.5.9-r16)[7] with default parameters. Alignments were converted from sequence alignment map (SAM) format to sorted, indexed binary alignment map (BAM) files (SAMtools version 0.1.18; http://sourceforge.net). The Picard tool was used to remove duplicate reads.

Efficiency of alignment and qualimap was used for evaluating alignment data (qualimap_v2.1.2, http://qualimap.bioinfo.cipf.es/). The Best Practices Workflow of Genome Analysis Toolkit (GATK version 2.7-4, http://www.broadinstitute.org) was used for improving the alignments and for genotype calling with recommended parameters[8]. BAM files were re-aligned with the GATK IndelRealigner, and base quality scores were re-calibrated by the GATK base quality recalibration tool. Genotypes were called at first with the GATK UG (version 2.7-4) and the GATK VariantRecalibrator tool was used to score variant calls by a machine-learning algorithm and to identify a set of high-quality variants using the Variant Quality Score Recalibration (VQSR) procedure. GATK was used to filter high-quality variants with hard filtering criteria (variant confidence score ≥30, mapping quality ≥40, read depth ≥5, and strand bias FS filter <60). At a later date, our exome data was also re-processed using the newer GATK algorithm HC (version v3.3). To be conservative, we decided to retain and evaluate both variant lists generated by HC and UG. These two tools are based on different algorithms[9]. HC in variable regions of the genome discards the existing mapping information and reassembles the reads (*de novo* assembly of haplotypes). UG uses a Bayesian genotype likelihood model and estimates the most likely genotype calls and simply looks for a coincident haplotype event in the reads. Both methods evaluate haplotypes using an affine gap penalty Pair Hidden Markov Model[10]. Variants were then annotated with the software snpEFF[11] (snpEff_v2_0_5, http://snpeff.sourceforge.net/download.html) and categorized into four classes (high, moderate, low and modifier) and the functional impact of coding variants was also predicted. Low impact variants were predicted by snpEFF and filtered out as they were synonymous coding and "assumed to be mostly harmless or unlikely to change protein behaviour" as decribed in manual (http://snpeff.sourceforge.net/SnpEff_manual.html). Sequence data were filtered against multiple databases, using annovar (http://annovar.openbioinformatics.org, version 2013Aug23) and Minor Allele Frequencies (MAF) of the called variants were compared against dbSNP 137 (ftp://ftp.ncbi.nih.gov/snp/) and 1000 Genomes Project, where we filtered against the European cohort (April 2002 release, ftp://ftp.1000genomes.ebi.ac.uk/vol1/ftp/). Then, variants were visualized with Integrative Genomics Viewer (IGV, version 2.3.36)[12]. The concordance between the genotypes of the variants identified through exome sequencing and bead SNP genotyping was evaluated using the PLINK[13] program. Concordance was of 96% and 94% for UG and HC, respectively.

Whole-exome sequencing data for all families have been deposited in the SRA database of NCBI (http://www.ncbi.nlm.nih.gov/sra) and are available under the following study accession No. SRP061415

Variant selection and Sanger Sequencing

Segregating variants in affected individuals were selected based on their scaled C-scores from the Combined Annotation Dependent Depletion (CADD) webserver (http://cadd.gs.washington.edu)[14]. The phred-like scores ("scaled C-scores") ranging from 1 to 99, are based on the rank of each variant relative to all possible 8.6 billion substitutions in the human reference genome (http://cadd.gs.washington.edu/info) and candidate variants were validated using Sanger Sequencing. The variants were validated at two different sites in the UK and in Italy. For the sequencing carried out in the UK, PCR primers were designed using the Primer 3 software. PCR amplification was carried out using the Clontech Advantage 2 PCR kit (Takara), following the manufacturer's recommended conditions. PCR products were purified prior to sequencing using exoSAP-IT (Affymetrix Inc), following the manufacturer's recommended conditions. For the sequencing carried out in Italy, forward and reverse PCR primers were designed for each candidate variant using the Primer Designer™ Tool (Life Technologies). PCR amplification was performed using AmpliTaq Gold® 360 DNA Polymerase (Life Technologies, Italia) and products were checked on agarose gels 2% and purified using the QIAquick PCR Purification kit (Qiagen). Purified products were sequenced in both forward and reverse directions on an ABI 3730xl DNA analyser (Applied Biosystems). Analysis of sequence data was carried out using the Chromas 2.01 software. Human reference sequences were retrieved from the UCSC Genome Browser.

REFERENCES

1 Gharavi AG, Yan Y, Scolari F*, et al.* IgA nephropathy, the most common cause of glomerulonephritis, is linked to 6q22-23. *Nat Genet* 2000; **26:** 354-7.

2 Bisceglia L, Cerullo G, Forabosco P*, et al.* Genetic heterogeneity in Italian families with IgA nephropathy: suggestive linkage for two novel IgA nephropathy loci. *Am J Hum Genet* 2006; **79:** 1130-4.

3 Schena FP, Cerullo G, Torres DD*, et al.* The IgA nephropathy Biobank. An important starting point for the genetic dissection of a complex trait. *BMC Nephrol* 2005; **6:** 14.

4 Sallustio F, Cox SN, Serino G*, et al.* Genome-wide scan identifies a copy number variable region at 3p21.1 that influences the TLR9 expression levels in IgA nephropathy patients. *Eur J Hum Genet* 2015; **23:** 940-8.

5 Abecasis GR, Cherny SS, Cookson WO, Cardon LR. Merlin--rapid analysis of dense genetic maps using sparse gene flow trees. *Nat Genet* 2002; **30:** 97-101.
6 Powell JE, Visscher PM, Goddard ME. Reconciling the analysis of IBD and IBS in complex trait studies. *Nat Rev Genet* 2010; **11:** 800-5.
7 Li H, Durbin R. Fast and accurate short read alignment with Burrows-Wheeler transform. *Bioinformatics* 2009; **25:** 1754-60.
8 DePristo MA, Banks E, Poplin R*, et al.* A framework for variation discovery and genotyping using next-generation DNA sequencing data. *Nat Genet* 2011; **43:** 491-8.
9 Pirooznia M, Kramer M, Parla J, Goes FS, Potash JB, McCombie WR, Zandi PP. Validation and assessment of variant calling pipelines for next-generation sequencing. *Hum Genomics* 2014; **8:** 14.
10 Krogh A, Brown M, Mian IS, Sjolander K, Haussler D. Hidden Markov models in computational biology. Applications to protein modeling. *J Mol Biol* 1994; **235:** 1501-31.
11 Cingolani P, Platts A, Wang le L*, et al.* A program for annotating and predicting the effects of single nucleotide polymorphisms, SnpEff: SNPs in the genome of Drosophila melanogaster strain w1118; iso-2; iso-3. *Fly (Austin)* 2012; **6:** 80-92.
12 Thorvaldsdottir H, Robinson JT, Mesirov JP. Integrative Genomics Viewer (IGV): high-performance genomics data visualization and exploration. *Brief Bioinform* 2013; **14:** 178-92.
13 Purcell S, Neale B, Todd-Brown K*, et al.* PLINK: A tool set for whole-genome association and population-based linkage analyses. *Am J Hum Genet* 2007; **81:** 559-75.
14 Kircher M, Witten DM, Jain P, O'Roak BJ, Cooper GM, Shendure J. A general framework for estimating the relative pathogenicity of human genetic variants. *Nat Genet* 2014; **46:** 310-5.

Supplementary Figure 1. Sixteen multiplex families included in the linkage study, the red bars represent 146 genotyped subjects. Squares and circles represent males and females, respectively; arrows indicate probands and the slash indicate deceased individuals. Filled and unfilled symbols indicate IgAN affected and unaffected individuals, respectively. The symbols with a dot indicate individuals who have not received or have discordant urinalysis. Symbols with a vertical line indicate individuals with documented urinary abnormalities (persistent microscopic hematuria and/or proteinuria). Families 1, 3, 4, were present in the first linkage study perfomed by Gharavi AG et al (Nat Genet 2000; 26: 354-7). Families 3, 4, 7, 15, 16, 206, 343, 344, 385, 386, 393 were also present in our previous linkage study perfomed by Bisceglia L et al (Am J Hum Genet 2006; 79: 1130-4). Families 14,17,36,483 were new and added exclusively for this study.

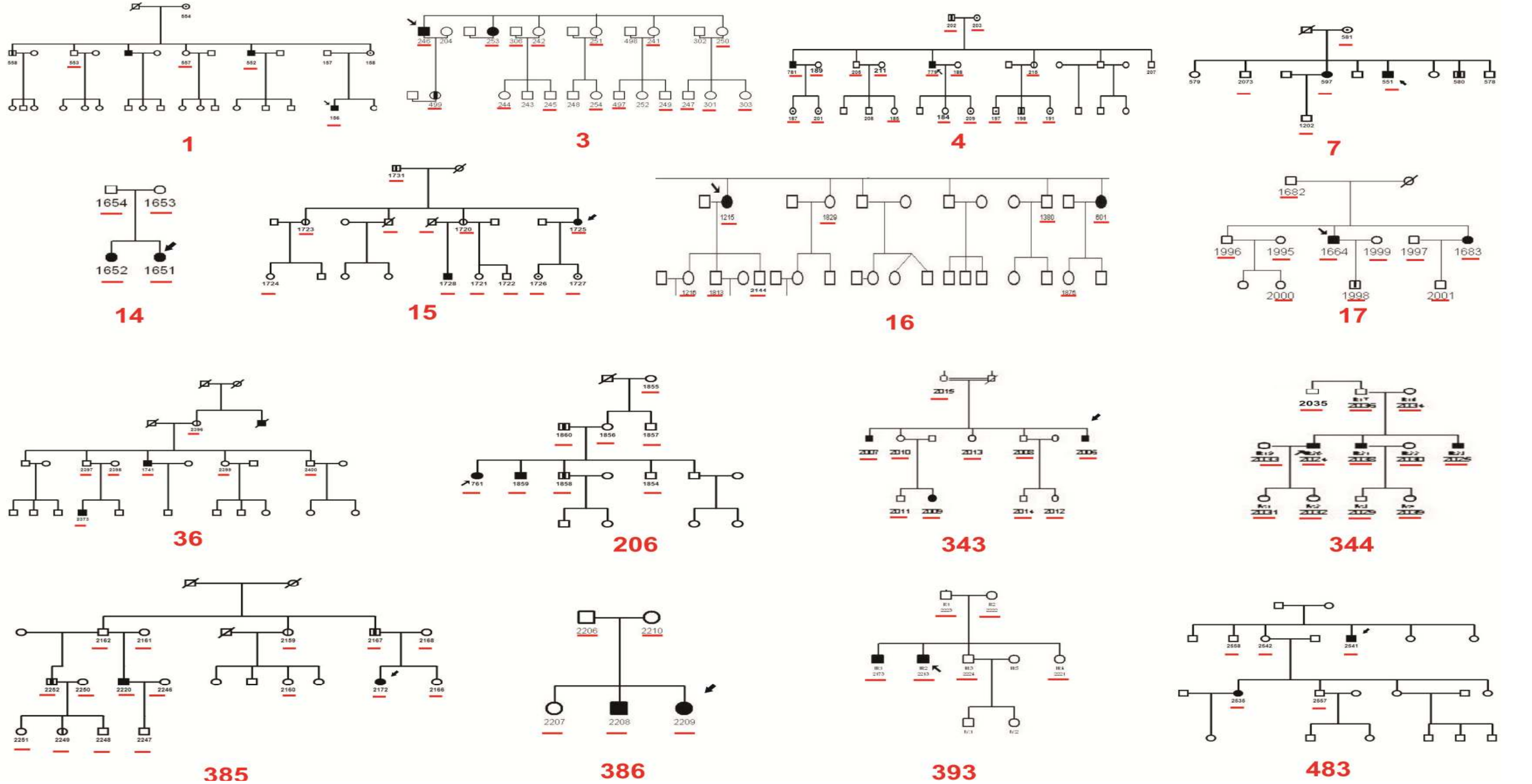

Supplementary Figure 2. Plot of LOD score statistics from NPL analysis for the chromosomes in which the score exceeded the 1.5 level.

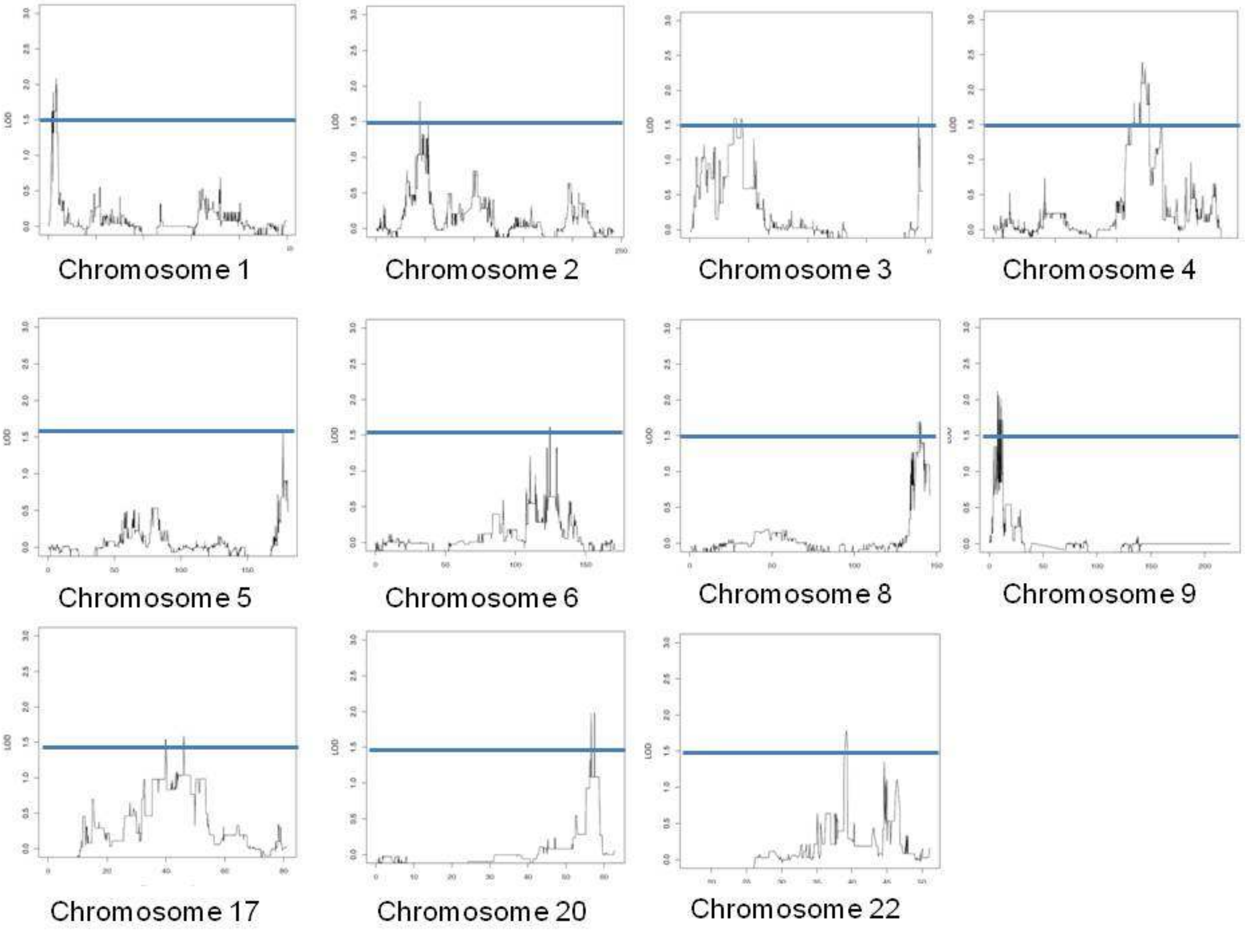

Supplementary Figure 3. Pedigrees included in the exome sequencing study

Eight pedigrees were included in the exome sequencing study. Squares and circles represent males and females, respectively; the arrows indicate probands and the slashes indicate deceased individuals. Filled and unfilled symbols indicate IgAN affected and unaffected individuals, respectively. The symbols with a dot indicate individuals who had not received or had intermittent microscopic hematuria. Symbols with a vertical line indicate individuals with urinary abnormalities. Horizontal grey lines show individuals on whom whole-exome sequencing has been performed.

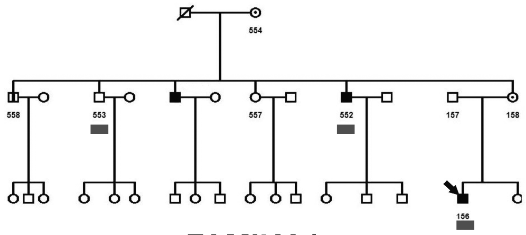

FAMILY 1

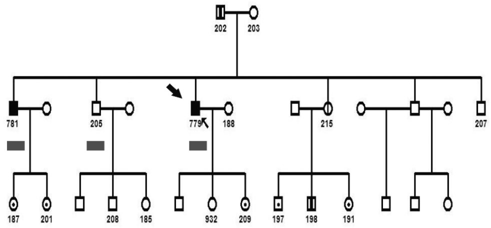

FAMILY 4

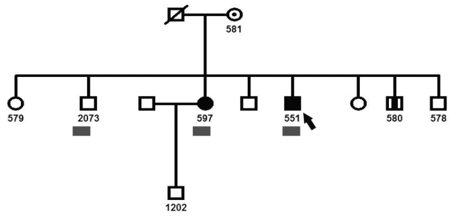

FAMILY 7

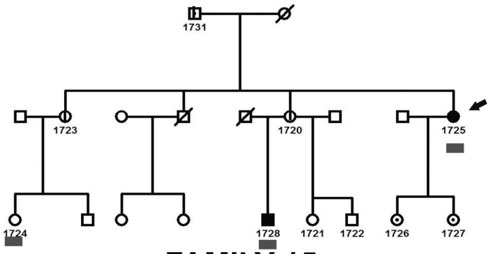

FAMILY 15

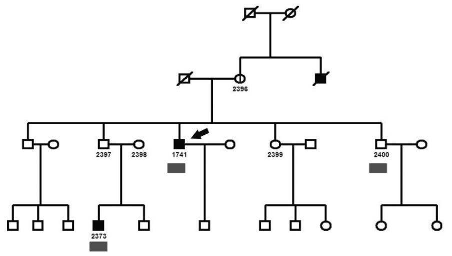

FAMILY 36

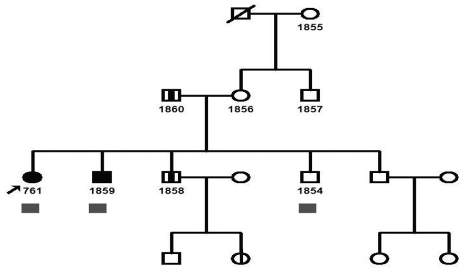

FAMILY 206

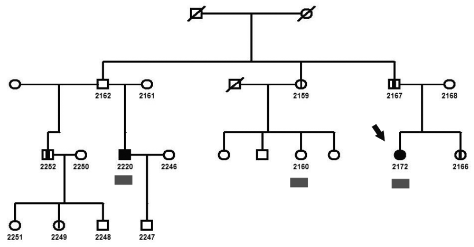

FAMILY 385

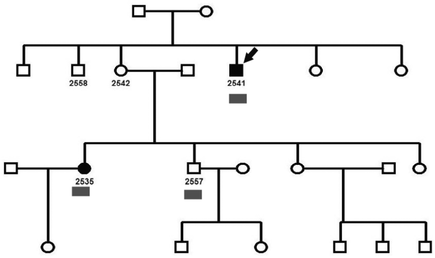

FAMILY 483

Supplementary Figure 4. Number of variants called by Unified Genotyper (UG) and Haplotype Caller (HC). Venn diagram depicts the number of unique and common variants when called by UG and UC (A). A total of 48598 variants have been called by HC and UG, of these 27724 (57%) were in common, 13735 (28%) were unique to HC and 7139 (15%) were unique to UG. (B)

A

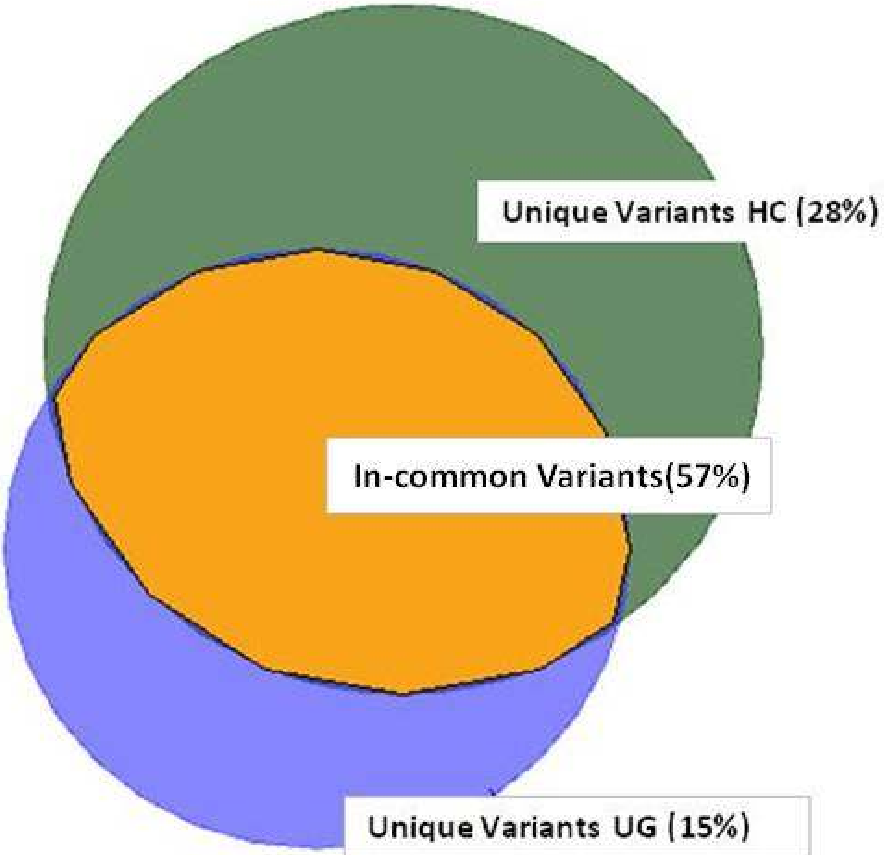

B

	TOTAL NUMBER	COMMON HC UG	UNIQUE HC	UNIQUE UG
variants No.	48598	27724	13735	7139
Variants %	100	57	28	15

Supplementary Figure 5. Percentage distributions of gene variantions as depicted in the output of SnpEff: Variant analysis. The percentage of variations within each gene region did not differ when called by Unified Genotyper (A) and Haplotype Caller (B).

A)

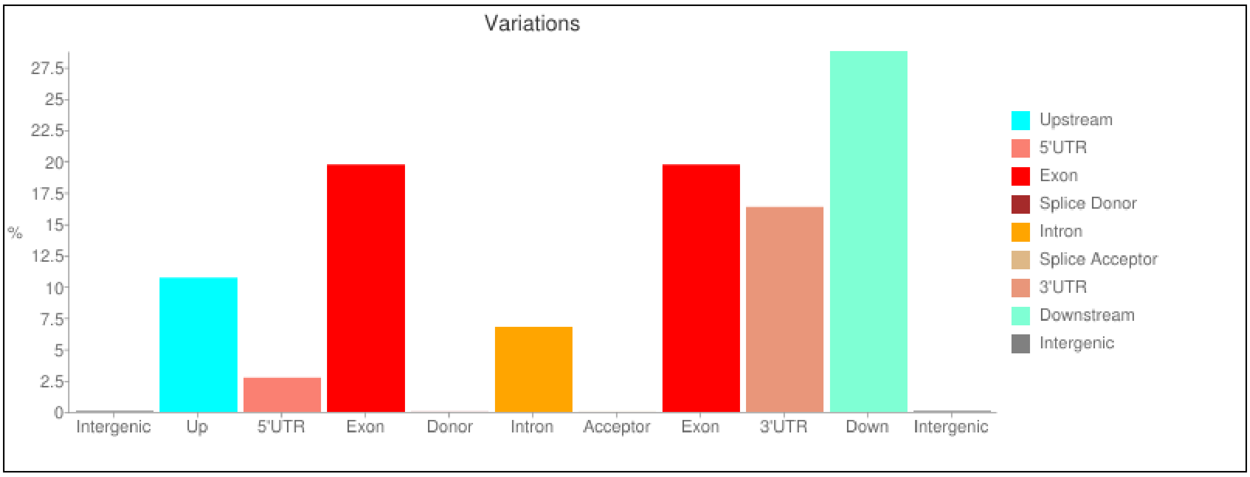

B)

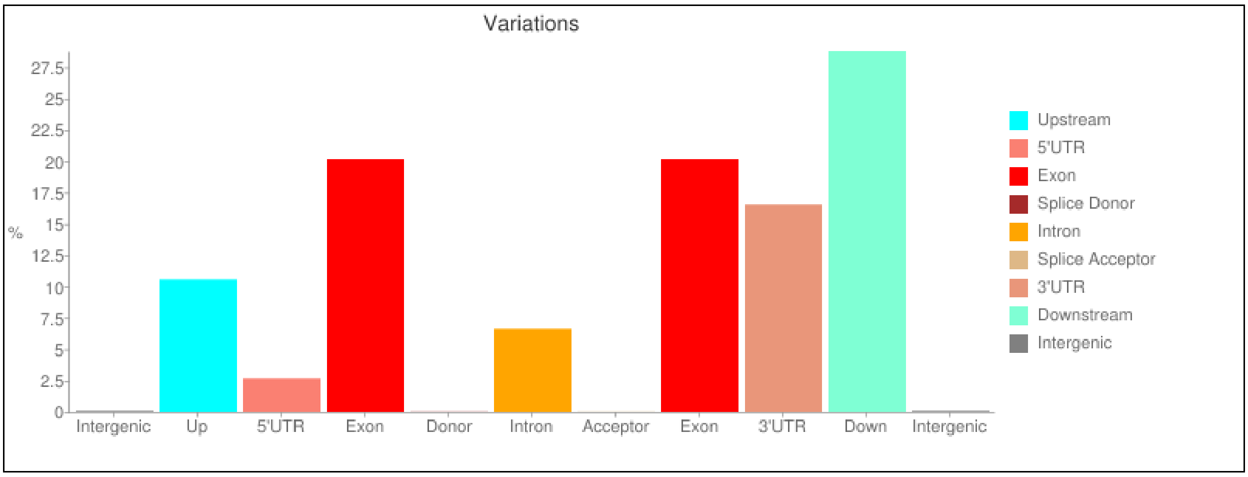

Supplementary Figure 6. The two variants predicted within the *BCLAF1* gene were excluded as they were actually determined by the presence of a deletion in a repeated (AAAAAC)n region (and not by a G/A substitution). Heterozygous deletion found in the repeated (AAAAAC)n region of the BCLAF1 gene. Deletion detected in the flipped reverse sequence of two affected individuals (552 ,156) and absent in the intra-familal control (553).

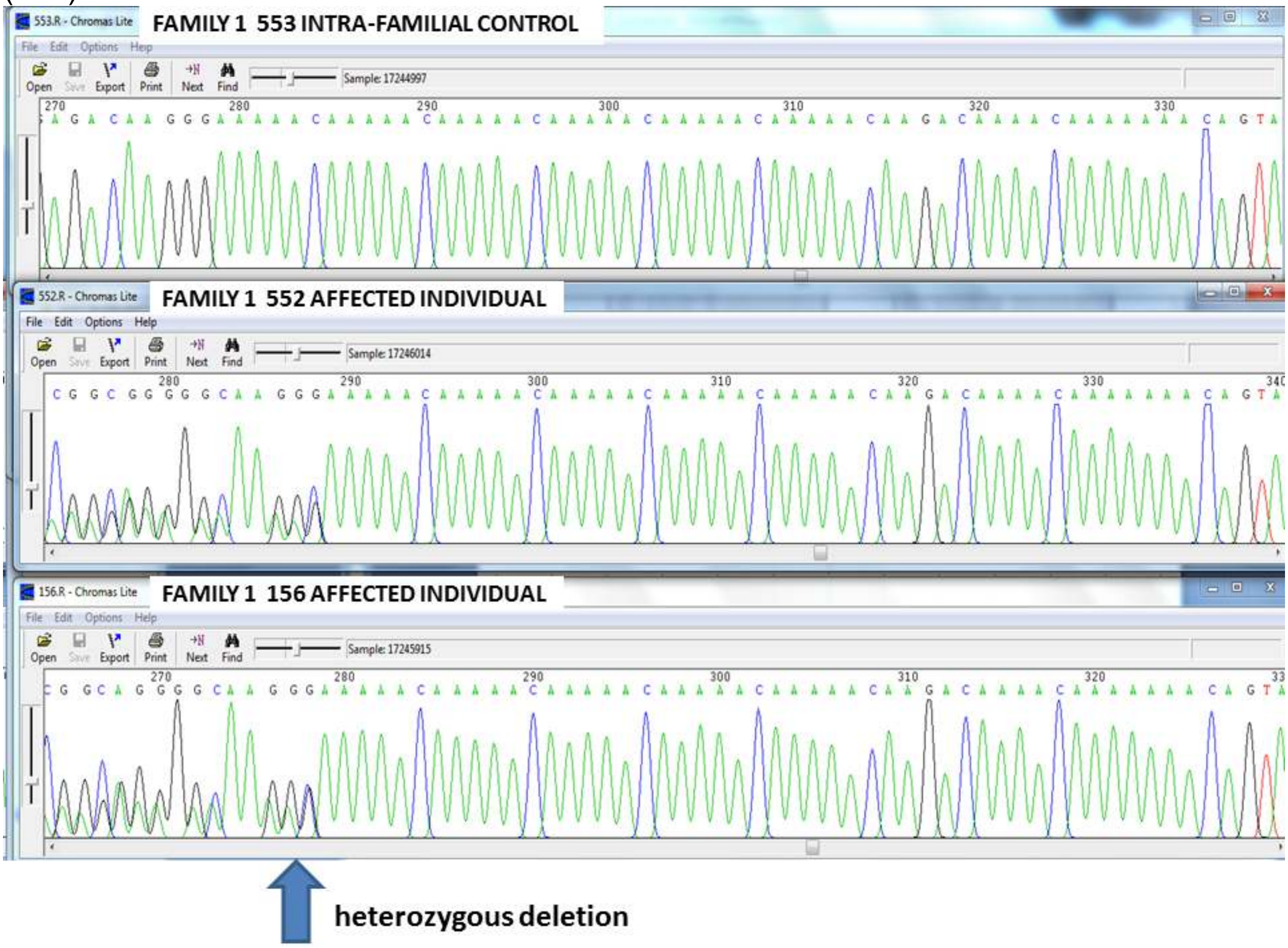

Supplementary Figure 7. We evaluated BCLAF1 complex genomic region in six unrelated HBD. We found that the heterozygous deletion in the repeated (AAAAAC)n region of the BCLAF1 gene was also found in three unrelated healthy blood donors (control1 , Control2, control3) and absent in another three unrelated healthy blood donors , for this reason we decided to exclude this variation from further analysis.

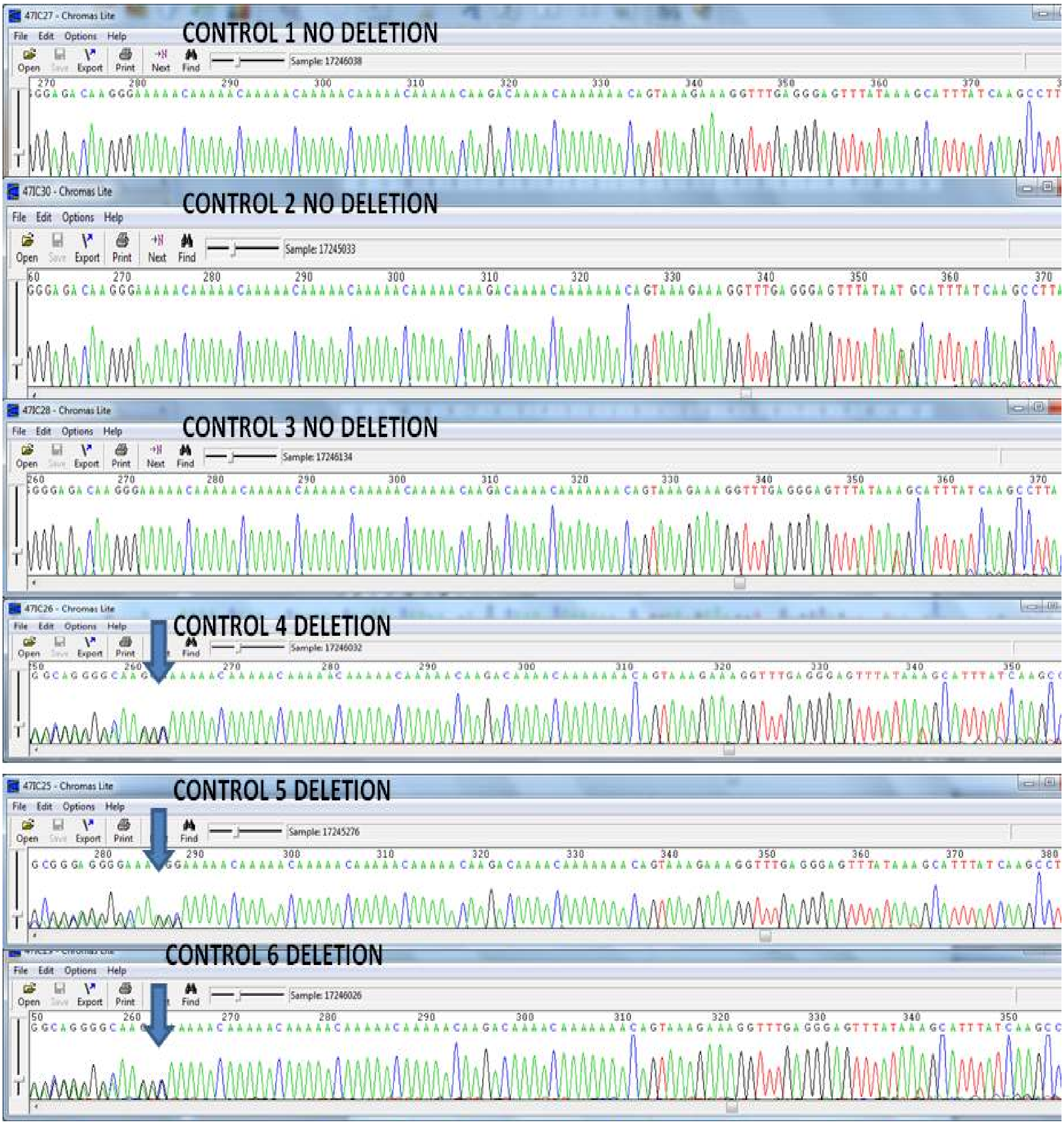

Supplementary Table 1 - Summary of the exome sequencing results

FAMILY ID	1			4			7			15			36			206			385			483		
SAMPLE ID	552	156	553	779	781	205	551	597	2073	1725	1728	1724	1741	2373	2400	761	1859	1854	2172	2220	2160	2535	2541	2557
STATUS	IgAN	IgAN	CNTR	IgAN	IgAN	CNTR	IgAN	IgAN	CNTR	IgAN	IgAN	CNTR	IgAN	IgAN	CNTR	IgAN	IgAN	CNTR	IgAN	IgAN	CNTR	IgAN	IgAN	CNTR
SEQUENCING AND MAPPING DATA																								
raw data Yield (Mbases)	4363	3658	4170	4084	745	2792	3829	4063	3135	2262	2838	5140	4201	3749	4210	2437	3699	4318	922	3871	4491	5940	3269	4159
n°Reads (M)	43.2	36.2	41.3	40.4	7.4	27.6	37.9	40.2	31.0	22.4	28.1	50.9	41.6	37.1	41.7	24.1	36.6	42.8	9.1	38.3	44.5	58.8	32.4	41.2
% mapped reads to Genome	98.59	98.29	98.59	98.58	98.55	98.48	98.29	96.52	96.13	97.28	97.15	98.2	98.4	98.4	98.58	98.42	98.54	98.46	96.56	97.3	98.27	98.17	97.33	97.38
EXOME CAPTURE																								
% mapped reads to target region [a]	44.25	42.27	42.95	42.64	44.56	42.93	42.27	43.61	43.09	42.75	42.88	43.24	42.49	42.58	43.88	44.75	44.35	42.15	45.85	43.76	42.3	42.95	44.66	43.39
mean coverage target region	26.54	22.36	24.56	23.87	4.54	16.44	22.36	24.11	18.44	13.22	16.76	24.32	23.79	21.35	24.63	14.5	21.74	24.29	5.72	23.15	24.98	24.24	19.93	24.59
mean mapping quality	50.16	50.00	50.17	50.14	50.00	49.99	50.13	49.65	49.18	49.75	49.16	49.75	50.00	50.10	50.04	49.97	50.00	50.00	49.08	49.00	50.72	50.22	49.93	50.08
VARIANT CALLING[b]																								
Unified Genotyper	45794	43065	45913	44714	24256	40897	45442	44435	41481	36509	40356	46305	50311	48924	50099	44443	48825	50437	23629	41296	43789	46940	40125	43406
Haplotype Caller	33647	30571	33568	32554	10681	27454	32936	32990	29383	23492	27782	35594	41168	39286	40793	33787	39417	41248	10774	29154	32012	36323	27361	31396

[a]Target region contains 62Mb of genomic DNA including exons, flanking 3'UTR , 5'UTR, predicted microRNA and other non coding RNA. b number of variants called for each individual by two distinct algorithms from the Genome Analysis Toolkit (GATK): Haplotype Caller and Unified Genotyper. Abbreviations: CNTR: Intra-familial control; Mbases: mega bases; M:Million.

Supplementary Table 2. Genomic intervals considered for variant filtration[a]

Locus	TOP LOD SCORE	[b]LEFT LOD=0	[c]Top Hit SNP	[b]RIGHT LOD=0	LEFT bp position	Top Hit Bp position	RIGHT bp position
1p36	2.1	rs3094315	rs6577472	rs7555884	752566	8206130	35373878
2p21	1.8	rs2702068	rs12613771	rs7600065	24689512	44490660	61019699
3p22	1.6	rs17440919	rs6550478	rs12054271	1238954	37526013	65027043
3q29	1.6	rs6778567	rs12629557	rs13083786	192611340	194057168	197811684
4q26	2.4	rs7693338	rs17006113	rs7658837	94766621	120714886	151117651
5q35	1.6	rs17738444	rs2731665	rs34865693	169444363	176857270	180695849
6q22	1.6	rs723318	rs2064687	rs13215778	107280314	124376164	146900563
8q24	1.7	rs7813493	rs11166903	rs35756786	130208039	140282987	146245372
9p24	2.1	rs10814410	rs11792985	rs10813550	46587	7664428	31351194
17q21	1.6	rs11651767	rs2256020	rs16977176	4073398	46050635	70321327
20q13	2.0	rs2206633	rs915039	rs11697347	42736729	56596617	62903830
22q13	1.8	rs5754779	rs2252528	rs9615919	34369696	39219932	48940644

[a]Human reference genome hg19. [b]SNPs where the LOD score dropped to zero.[c]SNP where the top LOD score was observed.

Supplementary Table 3. List of co-segregating gene variants identified by exome sequencing

FAMILY ID	GENE SYMBOL	GENE NAME	CHR	POS	REF	ALT	SNPEFF_EFFECT	LOCATION	TYPE	CADD SCORE V1.0
1	***CHD5***	chromodomain helicase DNA binding protein 5	1	6163696	G	A	DOWNSTREAM	Nucleus	enzyme	5
	FAM179A	family with sequence similarity 179, member A	2	29249757	AC	A	FRAME_SHIFT	Other	other	15
	BCLAF1	BCL2-associated transcription factor 1	6	136579552	A	G	DOWNSTREAM	Nucleus	transcription regulator	2
	IL22RA2	interleukin 22 receptor, alpha 2	6	137465358	C	T	UTR_3_PRIME	Plasma Membrane	transmembrane receptor	6
	FAM135B	family with sequence similarity 135, member B	8	139164674	T	C	NON_SYNONYMOUS_CODING=I682V	Other	enzyme	3
	NATD1	N-acetyltransferase domain containing 1	17	21145630	C	T	TRANSCRIPT	Other	other	4
	CDK12	cyclin-dependent kinase 12	17	37689446	C	T	UTR_3_PRIME	Nucleus	kinase	9
	MIRLET7BHG	MIRLET7B Host Gene	22	46453973	T	C	INTRON	Cytoplasm	microRNA	7
4	***ATRAID***	all-trans retinoic acid-induced differentiation factor	2	27439820	A	G	DOWNSTREAM	Nucleus	other	22
	STON1	stonin 1	2	48807759	A	G	UPSTREAM	Plasma Membrane	other	0
	RPUSD3	RNA pseudouridylate synthase domain containing 3	3	9880772	T	C	NON_SYNONYMOUS_CODING=T255A	Other	other	12
	SLC6A1	solute carrier family 6 (neurotransmitter transporter), member 1	3	11080144	G	A	DOWNSTREAM	Plasma Membrane	transporter	1
	TG	Thyroglobulin	8	133925492	C	T	STOP_GAINED=Q1454*	Extracellular Space	other	42
	IFNA21	interferon, alpha 21	9	21165905	C	T	UTR_3_PRIME	Extracellular Space	cytokine	5
	USP6	USP6 ubiquitin specific peptidase 6	17	5036210	T	G	NON_SYNONYMOUS_CODING=I67M	Cytoplasm	other	7
	INTERGENIC	-	17	36203079	C	T	-	-	-	3
7	***LDLRAP1***	low density lipoprotein receptor adaptor protein 1	1	25894878	C	G	DOWNSTREAM	Cytoplasm	transporter	4
	CAAP1	Caspase Activity And Apoptosis Inhibitor 1	9	26841936	C	A	DOWNSTREAM	other	other	7
	B4GALT5	UDP-Gal:betaGlcNAc beta 1,4-galactosyltransferase, polypeptide	20	48250578	T	C	UTR_3_PRIME	Cytoplasm	enzyme	11

		5								
15	***CFAP774***	cilia and flagella associated protein 74	1	1849195	C	T	DOWNSTREAM	Cytoplasm	other	4
	H6PD	hexose-6-phosphate dehydrogenase (glucose 1-dehydrogenase)	1	9326460	A	G	TRANSCRIPT	Cytoplasm	enzyme	4
	UBE4B	ubiquitination factor E4B	1	10190827	C	T	NON_SYNONYMOUS_CODING=R378C	Cytoplasm	other	35
	DFFA	DNA fragmentation factor, 45kDa, alpha polypeptide	1	10527277	G	C	NON_SYNONYMOUS_CODING=S137R	Nucleus	enzyme	17
	SLC6A6	solute carrier family 6 (neurotransmitter transporter), member 6	3	14528787	A	G	UTR_3_PRIME	Plasma Membrane	transporter	15
	JADE1	jade family PHD finger 1	4	129783008	T	A	NON_SYNONYMOUS_CODING=S365R	Nucleus	other	3
	SQSTM1	sequestosome 1	5	179264117	A	G	DOWNSTREAM	Cytoplasm	transcription regulator	6
36	***THADA***	thyroid adenoma associated	2	43455302	G	A	DOWNSTREAM	Other	other	15
	SETD5	SET domain containing 5	3	9515095	C	A	NON_SYNONYMOUS_CODING=S1026Y	Other	other	26
	PTPRG	protein tyrosine phosphatase, receptor type, G	3	62063912	G	A	NON_SYNONYMOUS_CODING=A199T	Plasma Membrane	phosphatase	22
	BCLAF1	BCL2-associated transcription factor 1	6	136579558	A	G	DOWNSTREAM	Nucleus	transcription regulator	2
	CYP11B2	cytochrome P450, family 11, subfamily B, polypeptide 2	8	143993975	C	T	NON_SYNONYMOUS_CODING=E457K	Cytoplasm	enzyme	14
	ZNF252	zinc finger protein 252, pseudogene	8	146199255	G	A	DOWNSTREAM	Other	other	2
	KIAA1432	RAB6A GEF complex partner 1	9	5775730	G	A	DOWNSTREAM	Cytoplasm	other	1
206	***CPLX2***	complexin 2	5	175310261	C	G	DOWNSTREAM	Cytoplasm	other	4
	PRSS3	protease, serine, 3	9	33799224	C	G	DOWNSTREAM	Extracellular Space	peptidase	1
	UBE2G1	ubiquitin-conjugating enzyme E2G 1	17	4173166	G	A	UTR_3_PRIME	Cytoplasm	enzyme	0.2
	TMEM107	transmembrane protein 107	17	8076656	A	G	DOWNSTREAM	Other	other	0.9
	USP22	Ubiquitin Specific Peptidase 22	17	20931986	G	T	NON_SYNONYMOUS_CODING=A126D	Extracellular Space	other	15
	SSH2	slingshot protein phosphatase 2	17	27958446	C	T	NON_SYNONYMOUS_CODING=G1229S	Cytoplasm	phosphatase	n.d

	THRA	thyroid hormone receptor, alpha	17	38233146	C	T	STOP_GAINED=R26*	Nucleus	ligand-dependent nuclear receptor	39
	CDC27	cell division cycle 27	17	45197967	A	G	DOWNSTREAM	Nucleus	other	9
	EME1	essential meiotic structure-specific endonuclease 1	17	48459319	A	G	DOWNSTREAM	Nucleus	other	3
	APOBEC3F	apolipoprotein B mRNA editing enzyme, catalytic polypeptide-like 3F	22	39448201	G	C	NON_SYNONYMOUS_CODING=E282D	Cytoplasm	enzyme	1
**385**	***CAMK2D***	calcium/calmodulin-dependent protein kinase II delta	4	114374628	T	A	DOWNSTREAM	Cytoplasm	kinase	10
	CHD5	chromodomain helicase DNA binding protein 5	1	6162250	G	GAC	DOWNSTREAM	Nucleus	enzyme	19
	ZNF696	zinc finger protein 696	8	144380221	G	A	DOWNSTREAM	Nucleus	other	2
**483**	***NBPF1***	neuroblastoma breakpoint family, member 15	1	16935213	G	A	TRANSCRIPT	Other	other	3
	SPTBN1	spectrin, beta, non-erythrocytic 1	2	54895817	ATTT	ATT, A	TRANSCRIPT	Plasma Membrane	other	n.d
	EDEM1	ER degradation enhancer, mannosidase alpha-like 1	3	5259973	A	G	UTR_3_PRIME	Cytoplasm	enzyme	5
	SDHAP1	succinate dehydrogenase complex, subunit A, flavoprotein pseudogene 1	3	195709098	G	A	DOWNSTREAM	Other	other	1
	TOM1L2	target of myb1-like 2 (chicken)	17	17748047	G	A	DOWNSTREAM	Cytoplasm	transporter	10
	ERAL1	Era-like 12S mitochondrial rRNA chaperone 1	17	27188606	A	C	DOWNSTREAM	Cytoplasm	other	17

Ref = reference allele; Alt = alternate allele; CADD SCORE: PHRED-like (-10*log10(rank/total)) scaled C-score ranking a variant relative to all possible substitutions of the human genome (8.6x10^9)

Supplementary Table 4. Left and right PCR primers designed to amplify the genomic region containing the variant of interest used for Sanger validation

GENE SYMBOL	VARIANT	LEFT PRIMER	RIGHT PRIMER
CDK12	17:g37689446c>t	CTTCAGCATCTTGGAGGGTAGT	AACACAAAGGCCCATGTCTGA
CHD5*	1:g6163696g>a	GGAGGGACCATCAGCCCTTG	TGGGTCAGGATTCACCAGCTT
FAM179A	2:g29249757ac>a	ATTCCCCTCAGCTCACATGG	AACATGTCTGTGCTGTCTCTGG
IL22RA2*	6:g137465358c>t	CTCCACAAAAGGACAAAAGGCAAA	GGTGCCTACAGAGACTATAGAGCTA
MIRLET7BHG*	22:g46453973t>c	GGAGATTGGTCCCCCTCCGTT	GGGTCACCCATGTCTCTACTGT
IFNA21	9:g21165905c>t	TACAAGAAAGCGAAAACGG	CCAGTTCCAGAAGGCTCA
ATRAID	2:g27439820a >g	GAGCCACAAGACCAGGAGCTGA	CGTGGTCTCCGCACTGCAAA
RPUSD3*	3:g9880772t>c	TGCACAGATAAGGTCTGGAGATGCT	CTAGCACCCCAACAGGAAAGAACAG
LDLRAP1*	1:g25894878c>g	CCTCGTGTCCTGCTAGCTGTC	CGCCCACGCCGCCTTAT
DFFA*	1:g10527277g>c	GGATGAACATTGTTGCAGGTTGTG	CAGGGTTGAAGTGGAAGAATGTG
JADE1	4:g129783008t>a	TTGCAGGGTTAATCCACG	CAGGGGCTTGTTGAAGTT
SLC6A6	3:g14528787a>g	TGGGCATTTGTGGTCATTTCA	GGAGACATGAAGGTTGAGCAT
SQSTM1*	5:g179264117a>g	GCAAAACAAGTGACATGAAGGG	AGTGCTGATGCCATTTAATTAGATTGT
UBE4B*	1:g10190827c>t	GCAGAAGATGATGTAAGTATAGTGGCT	CTTGCTCACCCTCACAGTGTA
CYP11B2*	8:g143993975c>t	CAGGTGTCAATCACACCATGC	CCATCCAGCTGAGGACCCTTT
SETD5*	3:g9515095c>a	GCGTGGCTTGGCTAGTGGTTTA	TGTTCCCATCAAGTGTTCCCATAG
PTPRG	3:g62063912g>a	TCTTTTACAATCCAGATGACTTTGACA	GGATTTCTGAGAGGGAAAGAGAGG
THADA*	2:g43455302g>a	ATCTAGCGCCCAGTGAGGCTAA	CTCATGATTCTGTGGCTCTCTGT
CDC27*	17:g45197967a>g	TCTGAATGTTAAAGGTGATCCCACA	TCTCCCTTTGTTGGAAAGTATCATG
THRA	17:g38233146c>t	GGTGGGAGGTAGAATGAGGAC	TACTGTTCCACATCCAGGTCC

UBE2G1*	17:g4173166g>a	GGCCAGAAAGCCACTCAGATCA	GGGATCTGACTCTTCAGCAAGCA
CAMK2D	4:g114374628t>a	GCTGGCTAGTAGTGTGTGAGA	GTGATGATGCAGAAGTGACCCT
CHD5	1:g6162250a>c	TTACAGGTTGTGGTGCATCAG	AAGGTGATTGTGTTGGCTACA
EDEM1*	3:g5259973a>g	CACTTTGCCTGTCACTCGAGCAA	GGCAAAGCACTGAGCAAAGCAA
B4GALT5*	20:g48250578t>c	TCGAGGCCTGGTGGACACAT	GAGCAGGTTCCTGCCCTTGAA
TOM1L2*	17:g17748047g>a	CCTGCTCAGTGCCTGGAGACT	CTGCACAGGAAGCAAGTATAGCC
ERAL1	17:g27188606a>c	AGGAAGCTGTGTGTGTCCAG	GGTAACGGTTTCCTTGCCATT
TG	8:g133925492c>t	CGCTTCACAGATCTGATCCAG	CAAATATGGGGCTCCTTCTGC
BCLAF1*	6:g136579552a>g	AAACTGACAGGATGGCACATGGT	TTACAAGGGTCCCTGTTGCATCA
BCLAF1*	6:g136579558a>g	AAACTGACAGGATGGCACATGGT	TTACAAGGGTCCCTGTTGCATCA
USP22*	17:g20931986g>t	GCCACAGACATGGCAGATACA	TACGAAACGTTGTGACAAAGGA
USP6*	17:g5036210t>g	CAGCAGAGACCTGACCCCAAGT	ATGGGTGCCTGTCCCCTGTTT
CAAP1*	9:g26841936c>a	TCCTGACAGCTTGGAATGTAAATG	GGCAATATAGTTGGATAGCCTGGAT

***** primers designed using the Sanger Primer Designer™ Tool (Life Technologies)

Supplementary Table 5. List of primers designed for SNP genotyping analysis using custom taqman® assays

GENE SYMBOL	VARIANT	LEFT PRIMER	RIGHT PRIMER	VIC_PROBE	FAM_PROBE
CAMK2D	4:g114374628t>a	AGTCACAGGAGGAACTTGCTTTTAT	TTAAGCTCTAGTTTGGACTTAGGTATCCT	TTTGCATTGTTTTAAGTTAG	TTGCATTGTTTAAAGTTAG
THRA	17:g38233146c>t	TGGAAAGAATCAGGCCTTGGG	CAGCCTCACCTGACATGCT	TTCTTTCTTTTTCACTTTCC	CTTTCTTTTTCGCTTTCC
MIRLET7BHG	22:g46423973t>c	CTGGCTGCTGGTACTAACTCTAAT	AGCTTCCAGGCCGTTTCC	TTGTCCCCAATCTTT	TTGTCCCCAGTCTTT
DFFA	1:g10527277g>c	GGATGAACATTGTTGCAGGTTGTG	CAGGGTTGAAGTGGAAGAATGTG	TCTGTCCAGCATCATC	CTGTCCAGGATCATC
SETD5	3:g9515095c>a	TGTGCAGGGATCCTCAGC	TGATATTCTCTTGAGGTCTGCAGTGA	CGAACTCCATCTTCCCCT	CGAACTCCATATTCCCCT
JADE1	4:g129783008t>a	GCCCAAAGCACAGCTCACATA	GCCTCCTCCCGGTTCTG	TTGCCAAGACTCTCC	TTGCCAAGTCTCTCC
UBE2G1	17:g4173166g>a	GTTGTTGATGGTTGGCACAAGTTT	CCACCAGTGCCTCATCAGT	AGAGAGGAATCGCCTCAC	AGAGAGGAATCACCTCAC

Supplementary Table 6. List of potential drugs targeting the network.

Drug Name	Targets	Actions
**11beta hydrocortisone acetate**	NR3C1	agonist
**3,5-diiodothyropropionic acid**	THRA	agonist
**acitretin**	RARA	agonist
**acitretin**	RARA	activator
**adapalene**	RARA	modulator
**afuresertib**	Akt	inhibitor
**alclometasone**	NR3C1	agonist
**alclometasone dipropionate**	NR3C1	agonist
**alitretinoin**	RARA	activator
**alitretinoin**	RARA	agonist
**amcinonide**	NR3C1	agonist
**amiodarone**	THRA	receptor antagonist activity
**archexin**	AKT1	binder
**ARQ 092**	AKT1	inhibitor
**arsenic trioxide**	RARA	chain breaker
**AT13148**	Akt	inhibitor
**AZD5363**	AKT1	inhibitor
**azelastine/fluticasone propionate [fluticasone]**	NR3C1	binder
**azelastine/fluticasone propionate [fluticasone]**	NR3C1	agonist
**bapineuzumab**	APP	antibody
**BAY1125976**	AKT1, AKT2	inhibitor
**beclomethasone**	NR3C1	agonist
**beclomethasone 17-monopropionate**	NR3C1	binder
**beclomethasone dipropionate**	NR3C1	agonist
**betamethasone**	NR3C1	agonist
**betamethasone acetate**	NR3C1	agonist
**betamethasone acetate/betamethasone phosphate [betamethasone acetate]**	NR3C1	agonist
**betamethasone benzoate**	NR3C1	agonist
**betamethasone dipropionate**	NR3C1	agonist
**betamethasone dipropionate/calcipotriene [betamethasone dipropionate]**	NR3C1	agonist
**betamethasone phosphate**	NR3C1	agonist
**betamethasone valerate**	NR3C1	agonist
**betamethasone/clotrimazole [betamethasone]**	NR3C1	agonist
**bortezomib/dexamethasone [dexamethasone]**	NR3C1	agonist
**bortezomib/dexamethasone/doxorubicin [dexamethasone]**	NR3C1	agonist
**bortezomib/dexamethasone/lenalidomide [dexamethasone]**	NR3C1	agonist
**bortezomib/dexamethasone/rituximab [dexamethasone]**	NR3C1	agonist
**bortezomib/dexamethasone/thalidomide [dexamethasone]**	NR3C1	agonist
**bortezomib/dexamethasone/thalidomide [thalidomide]**	NFKB1	antagonist
**bortezomib/prednisone [prednisone]**	NR3C1	agonist
**bortezomib/thalidomide [thalidomide]**	NFKB1	antagonist

**budesonide**	NR3C1	inhibitor
**budesonide**	NR3C1	antagonist
**budesonide/formoterol [budesonide]**	NR3C1	inhibitor
**budesonide/formoterol [budesonide]**	NR3C1	antagonist
**cabazitaxel/prednisone [prednisone]**	NR3C1	agonist
**carfilzomib/dexamethasone/lenalidomide [dexamethasone]**	NR3C1	agonist
**carfilzomib/dexamethasone/rituximab [dexamethasone]**	NR3C1	agonist
**carmustine/prednisone [prednisone]**	NR3C1	agonist
**CAT-354**	IL13	antibody
**chlorambucil/mitoxantrone/prednisone [prednisone]**	NR3C1	agonist
**chlorambucil/mitoxantrone/prednisone/rituximab [prednisone]**	NR3C1	agonist
**ciclesonide**	NR3C1	agonist
**ciprofloxacin/dexamethasone [dexamethasone]**	NR3C1	agonist
**ciprofloxacin/hydrocortisone [hydrocortisone]**	NR3C1	agonist
**clobetasol propionate**	NR3C1	agonist
**clocortolone**	NR3C1	agonist
**clocortolone pivalate**	NR3C1	agonist
**cortisone acetate**	NR3C1	agonist
**cyclophosphamide/daunorubicin/imatinib/prednisone/vincristine [prednisone]**	NR3C1	agonist
**cyclophosphamide/dexamethasone/rituximab [dexamethasone]**	NR3C1	agonist
**cyclophosphamide/dexamethasone/thalidomide [dexamethasone]**	NR3C1	agonist
**cyclophosphamide/etoposide/prednisone/rituximab/vincristine [prednisone]**	NR3C1	agonist
**cyclophosphamide/etoposide/prednisone/vincristine [prednisone]**	NR3C1	agonist
**cyclophosphamide/gemcitabine/prednisolone/rituximab/vincristine [prednisolone]**	NR3C1	agonist
**cyclophosphamide/mitoxantrone/prednisone/rituximab/vincristine [prednisone]**	NR3C1	agonist
**cyclophosphamide/mitoxantrone/prednisone/vincristine [prednisone]**	NR3C1	agonist
**cyclophosphamide/prednisolone/rituximab/vincristine [prednisolone]**	NR3C1	agonist
**cyclophosphamide/prednisolone/vincristine [prednisolone]**	NR3C1	agonist
**cyclophosphamide/prednisone [prednisone]**	NR3C1	agonist
**cyclophosphamide/prednisone/rituximab [prednisone]**	NR3C1	agonist
**cyclophosphamide/prednisone/rituximab/vincristine [prednisone]**	NR3C1	agonist
**cyclophosphamide/prednisone/vincristine [prednisone]**	NR3C1	agonist
**cytarabine/dexamethasone [dexamethasone]**	NR3C1	agonist
**cytarabine/dexamethasone/methotrexate [dexamethasone]**	NR3C1	agonist
**cytarabine/dexamethasone/oxaliplatin/rituximab [dexamethasone]**	NR3C1	agonist
**daunorubicin/tretinoin [tretinoin]**	RARA	agonist
**denosumab/levothyroxine [levothyroxine]**	THRA	agonist
**desonide**	NR3C1	agonist
**desoximetasone**	NR3C1	agonist

**dexamethasone**	NR3C1	agonist
**dexamethasone 21-acetate**	NR3C1	agonist
**dexamethasone phosphate**	NR3C1	agonist
**dexamethasone/fludarabine phosphate/mitoxantrone [dexamethasone]**	NR3C1	agonist
**dexamethasone/fludarabine phosphate/mitoxantrone/rituximab [dexamethasone]**	NR3C1	agonist
**dexamethasone/lenalidomide [dexamethasone]**	NR3C1	agonist
**dexamethasone/lenalidomide/sorafenib [dexamethasone]**	NR3C1	agonist
**dexamethasone/pomalidomide [dexamethasone]**	NR3C1	agonist
**dexamethasone/thalidomide [dexamethasone]**	NR3C1	agonist
**dexamethasone/thalidomide [thalidomide]**	NFKB1	antagonist
**dexanabinol**	NFkB (complex)	inhibitor
**dextrothyroxine**	THRA	agonist
**diflorasone diacetate**	NR3C1	agonist
**difluprednate**	NR3C1	agonist
**docetaxel/hydrocortisone [hydrocortisone]**	NR3C1	agonist
**docetaxel/prednisone [prednisone]**	NR3C1	agonist
**doxorubicin/tretinoin [tretinoin]**	RARA	agonist
**enzastaurin**	AKT1, AKT2, AKT3	inhibitor
**etretinate**	RARA	activator
**etretinate**	RARA	agonist
**everolimus/prednisone [prednisone]**	NR3C1	agonist
**florbetaben F**	APP	binder
**florbetapir F18**	APP	binder
**fludrocortisone acetate**	NR3C1	agonist
**flunisolide**	NR3C1	agonist
**fluocinolone acetonide**	NR3C1	agonist
**fluocinonide**	NR3C1	agonist
**fluorometholone**	NR3C1	agonist
**fluorometholone acetate**	NR3C1	agonist
**flurandrenolide**	NR3C1	agonist
**fluticasone**	NR3C1	binder
**fluticasone**	NR3C1	agonist
**fluticasone furoate**	NR3C1	agonist
**fluticasone furoate**	NR3C1	receptor antagonist activity
**fluticasone furoate/vilanterol [fluticasone furoate]**	NR3C1	receptor antagonist activity
**fluticasone furoate/vilanterol [fluticasone furoate]**	NR3C1	agonist
**fluticasone/salmeterol [fluticasone]**	NR3C1	binder
**fluticasone/salmeterol [fluticasone]**	NR3C1	agonist
**formoterol/mometasone furoate [mometasone furoate]**	NR3C1	agonist
**formoterol/mometasone furoate [mometasone furoate]**	NR3C1	inhibitor
**GSK2141795**	AKT1	inhibitor
**halcinonide**	NR3C1	agonist

**halobetasol propionate**	NR3C1	agonist
**hydrocortisone**	NR3C1	agonist
**hydrocortisone buteprate**	NR3C1	agonist
**hydrocortisone butyrate**	NR3C1	agonist
**hydrocortisone cypionate**	NR3C1	agonist
**hydrocortisone phosphate**	NR3C1	agonist
**hydrocortisone succinate**	NR3C1	agonist
**hydrocortisone valerate**	NR3C1	agonist
**hydrocortisone/mitoxantrone [hydrocortisone]**	NR3C1	agonist
**hydrocortisone/prednisone [prednisone]**	NR3C1	agonist
**idarubicin/tretinoin [tretinoin]**	RARA	agonist
**ipatasertib**	Akt, AKT1	inhibitor
**isotretinoin**	RARA	modulator
**L-asparaginase/prednisone/vincristine [prednisone]**	NR3C1	agonist
**L-triiodothyronine**	THRA	agonist
**L-triiodothyronine**	THRA	stimulator
**levothyroxine**	THRA	stimulator
**levothyroxine**	THRA	agonist
**loteprednol etabonate**	NR3C1	agonist
**LY2780301**	AKT1	inhibitor
**MEDI4736**	CD274	binder
**medrysone**	NR3C1	agonist
**methylprednisolone**	NR3C1	agonist
**methylprednisolone acetate**	NR3C1	agonist
**methylprednisolone succinate**	NR3C1	agonist
**methylprednisolone/rituximab [methylprednisolone]**	NR3C1	agonist
**miconazole**	NR3C1	antagonist
**mifepristone**	NR3C1	antagonist
**mitoxantrone/prednisone [prednisone]**	NR3C1	agonist
**MK2206**	AKT1	protein kinase inhibitor activity
**mometasone furoate**	NR3C1	agonist
**mometasone furoate**	NR3C1	inhibitor
**MPDL3280A**	CD274	binder
**MPT0E028**	AKT1	inhibitor
**MSB0010718C**	CD274	antibody
**MSC2363318A**	Akt	inhibitor
**NF-kappaB decoy**	RELA	binder
**NF-kappaB inhibitor**	NFkB (complex)	inhibitor
**octreotide/prednisone [prednisone]**	NR3C1	agonist
**ONC-201**	Akt	inhibitor
**ORG 34517**	NR3C1	antagonist
**perifosine**	AKT1	inhibitor
**prednicarbate**	NR3C1	agonist
**prednisolone**	NR3C1	agonist
**prednisolone acetate**	NR3C1	agonist

**prednisolone phosphate**	NR3C1	agonist
**prednisolone tebutate**	NR3C1	agonist
**prednisone**	NR3C1	agonist
**prednisone/somatotropin [prednisone]**	NR3C1	agonist
**prednisone/thalidomide [prednisone]**	NR3C1	agonist
**prednisone/thalidomide [thalidomide]**	NFKB1	antagonist
**PRI-724**	CTNNB1	antagonist
**ras inhibitor**	Ras	inhibitor
**rimexolone**	NR3C1	agonist
**rituximab/thalidomide [thalidomide]**	NFKB1	antagonist
**SR-13668**	Akt	inhibitor
**tamibarotene**	RARA	agonist
**tazarotene**	RARA	agonist
**tazarotene**	RARA	modulator
**thalidomide**	NFKB1	antagonist
**tretinoin**	RARA	agonist
**triamcinolone**	NR3C1	agonist
**triamcinolone acetonide**	NR3C1	agonist
**triflusal**	NFKB1	antagonist
